# Development of a rapid, simple, and sensitive point-of-care technology platform utilizing ternary NanoLuc

**DOI:** 10.3389/fmicb.2022.970233

**Published:** 2022-10-26

**Authors:** Emily A. Torio, Valerie T. Ressler, Virginia A. Kincaid, Robin Hurst, Mary P. Hall, Lance P. Encell, Kristopher Zimmerman, Stuart K. Forsyth, William M. Rehrauer, Molly A. Accola, Chia-Chang Hsu, Thomas Machleidt, Melanie L. Dart

**Affiliations:** ^1^Promega Corporation, Madison, WI, United States; ^2^Department of Pathology and Laboratory Medicine, School of Medicine and Public Health, University of Wisconsin-Madison, Madison, WI, United States; ^3^University of Wisconsin Hospital and Clinics, Clinical Laboratories, Madison, WI, United States

**Keywords:** point-of-care, bioluminescence, NanoLuc complementation, SARS-CoV-2 antigen, rapid testing

## Abstract

Point-of-care tests are highly valuable in providing fast results for medical decisions for greater flexibility in patient care. Many diagnostic tests, such as ELISAs, that are commonly used within clinical laboratory settings require trained technicians, laborious workflows, and complex instrumentation hindering their translation into point-of-care applications. Herein, we demonstrate the use of a homogeneous, bioluminescent-based, split reporter platform that enables a simple, sensitive, and rapid method for analyte detection in clinical samples. We developed this point-of-care application using an optimized ternary, split-NanoLuc luciferase reporter system that consists of two small reporter peptides added as appendages to analyte-specific affinity reagents. A bright, stable bioluminescent signal is generated as the affinity reagents bind to the analyte, allowing for proximity-induced complementation between the two reporter peptides and the polypeptide protein, in addition to the furimazine substrate. Through lyophilization of the stabilized reporter system with the formulated substrate, we demonstrate a shelf-stable, all-in-one, add-and-read analyte-detection system for use in complex sample matrices at the point-of-care. We highlight the modularity of this platform using two distinct SARS-CoV-2 model systems: SARS-CoV-2 N-antigen detection for active infections and anti-SARS-CoV-2 antibodies for immunity status detection using chemically conjugated or genetically fused affinity reagents, respectively. This technology provides a simple and standardized method to develop rapid, robust, and sensitive analyte-detection assays with flexible assay formatting making this an ideal platform for research, clinical laboratory, as well as point-of-care applications utilizing a simple handheld luminometer.

## Introduction

Point-of-care testing (POCT) is an essential tool for the rapid detection of analytes at the site of the patient enabling quick, actionable, informed medical decisions for improved disease management. This form of decentralized diagnostic analysis allows for testing at the bedside, outpatient clinics, and even over the counter as direct-to-consumer tests (Luppa et al., 2011; Tolan, 2017). There is a continuous and growing need for POCTs as they offer rapid turnaround times and streamlined workflows, thereby reducing the need for multiple patient visits, improving medication adherence and antibiotic stewardship, and aiding in the containment of infectious diseases (Patzer et al., 2018; Cooke et al., 2020; HealthEuropa, 2021).

The COVID-19 pandemic, caused by the SARS-CoV-2 virus, has exacerbated the need for POCTs while highlighting their utility both in the clinic and in the hands of a patient. This demand is evidenced by the widespread use of COVID-19 rapid antigen tests (RATs) which have been employed as a useful screening tool for telemedicine visits and drive-thru clinics in addition to their utility in contact tracing (Allen et al., 2020; Chau et al., 2020; Ngo et al., 2020). While the ongoing COVID-19 global pandemic has emphasized the importance of POCTs, these technologies serve an essential role in addressing a variety of public health and safety issues. For example, POCTs are proven tools for the detection of potential contaminants, pathogens, and biothreats in agricultural water sources, food production facilities, and public health settings (Louie et al., 2009; Malcata et al., 2020; Hu et al., 2021).

Analyte tests are used for the detection of biomolecules from a wide array of sample types and matrices. Heterogeneous immunoassays, such as ELISAs, remain the gold standard for analyte quantitation and are widely used in clinical laboratory settings due to their sensitivity, specificity, and modularity. Unfortunately, these assays have workflow limitations, requiring skilled technicians, multiple wash steps, long incubation times, and specialized equipment, which makes it difficult to transition the technology from lab-based to point-of-care formats which require speed, simplicity, and reagent stability (Kozel and Burnham-Marusich, 2017). The dominant format for POCT is lateral flow immunoassay (LFIA) that is configured to mimic a heterogeneous ELISA-like workflow by use of capillary action to separate bound from unbound material to capture the analyte-specific detection signal without the need for multiple wash steps (Koczula and Gallotta, 2016). The majority of LFIAs rely on either colorimetric reporter systems that can be read with the naked eye or fluorescence-based systems that require instrumentation for detection (Di Nardo et al., 2021). Despite the extensive use of POCTs, they continue to be plagued by widespread issues including low sensitivity, sample-matrix interferences, rigid analyses time frames, manufacturing difficulties, format inflexibility, and multiple reagent and material components (St John and Price, 2014; Shaw, 2016; Dalton, 2021). Many of these limitations are inherently linked to the colorimetric and fluorescence-based reporters that dominate the point-of-care space indicating that new chemistries must be developed to overcome these challenges (Koczula and Gallotta, 2016; Roy et al., 2022).

Bioluminescence-based reporter assays have shown distinct advantages over other reporter chemistries including low intrinsic background and broad dynamic range (Allard, 2008). This makes bioluminescence potentially well suited for use with complex sample matrices, like blood and serum, as the background is not affected by autofluorescence as seen with fluorescent detection methods, thus improving sensitivity (Fan and Wood, 2007; Suzuki and Nagai, 2017). Additionally, bioluminescence-based reporters do not require complex instrumentation for analyses since the readout is simply capturing the total light emission which negates the need for filters, mirrors, and external excitation light sources (Tung et al., 2016). These attributes make bioluminescence a promising readout modality for POCT formats that have historically suffered from poor sensitivity when compared to clinical lab-based assays (Hwang et al., 2020; Shaw, 2021).

NanoLuc (Nluc) is a small engineered 19 kDa luciferase. Its brightness and compact thermodynamically stable 10-strand β-barrel structure make Nluc an ideal candidate to meet the challenges faced during POCT development (Hall et al., 2012). Recently reported POCT biosensors such as luciferase-based indicators of drugs (LUCIDS) and luminescent antibody sensors have been developed for bioluminescent resonance energy transfer (BRET) based detection of analytes. The ratiometric nature of BRET measurements allows for robust quantification of analytes independent of bioluminescent signal intensity and sample volume.

However, these BRET-based platforms tend to be complex and require extensive re-design and optimization of biosensors for each analyte of interest which makes rapid prototyping and development challenging (Arts et al., 2017; Xue et al., 2017; Tenda et al., 2018; Ni et al., 2019; Tomimuro et al., 2020; Ni et al., 2021). Additionally, poor environmental stability of principal assay components, including coelenterazine-based luciferase substrates restrict the use of these platforms to cold storage-dependent designs which significantly restricts the range of potential POCT applications (Hawkins et al., 2002).

Split versions of Nluc have been used as binary or ternary complementation reporters for the sensitive detection of analytes using several configurations (Dixon et al., 2016, 2017; Schwinn et al., 2018; Elledge et al., 2020; Hwang et al., 2020; Hall et al., 2021; Kainulainen et al., 2021; Ni et al., 2021; Yao et al., 2021; Kincaid et al., 2022). These examples highlighted the benefits of using bioluminescence for analyte detection providing sensitive assays with simplified workflows. However, to meet the requirements needed for the POCT setting, the assay must be able to maintain performance paired with thermal and chemical stability for extended shelf-life in various environments. Previously reported assays that were based on the binary Nluc system or earlier versions of the ternary Nluc system were shown to carry liabilities in reporter stability as well as the inability to create the high concentration of reagents and substrate needed for a one-pot lyophilized cake (Dixon et al., 2017; Ohmuro-Matsuyama and Ueda, 2018).

The ternary Nluc technology combined with formulated substrate enabled the generation of shelf-stable lyophilized single-reagent homogeneous immunoassays for the sensitive detection analytes in clinical samples (Hall et al., 2021). The ternary Nluc cleaved an additional peptide piece from the binary LgBiT piece to form a new 17 kDa, 8-strand polypeptide piece (LgTrip) and two reporter peptides (β9 and β10). Since the three pieces have low affinities toward one another, complementation of the functional luciferase is driven quantitatively by analyte-induced interactions bringing these three pieces within a close enough proximity to interact, thus increasing simplicity and reducing the need for wash steps without affecting sensitivity as the background remains low (Hall et al., 2021). This system has the additional advantage over the binary system in that the peptide-tagged affinity reagent concentrations are independent from the light generation allowing to work under saturating conditions of the LgTrip reagent thus maximizing assay performance (Dixon et al., 2016; Hwang et al., 2020; Hall et al., 2021).

The benefits of the ternary Nluc reporter system make it well suited to address current limitations with POCTs. In this study, we utilized a further evolved version of the ternary Nluc system with improved strand 9 peptide solubility and enhanced LgTrip stability to design assays specifically formatted for rapid add-and-read POCT. Using COVID-19 as a model system, we utilize the modularity of this platform for development of sensitive and specific assays to detect either SARS-CoV-2 antigen proteins or anti-SARS-CoV-2 antibodies in clinical samples with POC-compatible formats. These assays provide a large dynamic range, exceeding three orders of magnitude, with a rapid 15-min readout. We showcase the flexibility of the reporter peptides by attaching them to the recognition elements *via* either chemical conjugation or as genetic fusion. We also demonstrate that these assays are highly adaptable and scalable to different assay formats as demonstrated here using both, a moderate-high-throughput plate-based format as well as nasal swab POCT without the need for changing the reagent components. In summary, this platform allows for the fast development of simple, add-and-read analyte-detection assays in a variety of formats relevant for point-of-care applications across a broad range of clinically relevant analytes.

## Materials and methods

### Antibody labeling

The following antibody pairs were used for the SARS-CoV-2 receptor binding domain (RBD) and nucleocapsid model systems: (1) Chimeric anti-human SARS-CoV/SARS-CoV-2 Spike monoclonal antibodies (mAbs) Cat: 40150-D002 and Cat: 40150-D003 (Sino Biological) and (2) Mouse anti-human SARS-CoV-2 Nucleocapsid mAbs Cat: 9547 and 9,548 (Meridian Bioscience). Labeling of the antibodies with the HaloTag-peptide fusions was performed as previously described (Hall et al., 2021). Antibodies were buffer exchanged 3X into 10 mM sodium bicarbonate buffer (pH 8.5) using Zeba spin desalting columns (Thermo Fisher). Antibodies were then incubated with 200 μM amine-reactive HaloTag Succinimidyl Ester (04) Ligand (Promega) for 2 h with orbital shaking (1,000 rpm) at 22°C. Unreacted ligand was removed with two passes through Zeba spin columns in Phosphate Buffered Saline (PBS). Antibodies were then incubated with 30 μM of the HaloTag-peptide 840 or HaloTag-*VS*-HiBiT fusion protein overnight with shaking at 4°C. HaloLink Resin (Promega) was used to remove excess HaloTag fusions from the reactions and characterization of antibody labeling was carried out using SDS-PAGE.

### Solution-based SARS-CoV-2 RBD or N immunoassay on GloMax

A 2X master mix stock solution containing 60 ng/ml β9-labeled anti-SARS-CoV-2 spike RBD antibody D003, 120 ng/ml β10-labeled anti- SARS-CoV-2 spike RBD antibody D002, and 2 μM LgTrip protein was prepared in assay buffer consisting of 0.01% Blocker BSA (Thermo Fisher) in PBS (PBSB), and 50 μl/well was dispensed in solid, white, nonbinding surface (NBS) 96-well plates (Costar). Either a 2× solution containing recombinant SARS-CoV-2 Spike or heat-inactivated virus for dose-response curve generation was prepared, and 50 μl/ well was added to the wells containing the master mix. Plates were incubated for 90 min at ambient temperature prior to the addition of 100 μl/well of a 30-fold dilution of Fz (Nano-Glo Live Cell Substrate; Promega N205) in PBSB. Assays were read on a GloMax Discover Multimode Microplate Reader (Promega) collecting total luminescence using kinetic or endpoint reads, depending on the experimental design. Variations on this methodology, including shorter incubation times, were examined as part of the optimization process. The same method was carried out for the nucleocapsid model system by using 60 ng/ml β9-labeled anti-SARS-CoV-2 N mAb Cat. 9,547, 120 ng/ml β10-labeled anti-SARS-CoV-2 N mAb Cat. 9,548, and 2 μM LgTrip and creating 2× titrations of recombinant CoV N-proteins or heat-inactivated SARS-CoV-2 variants (a comprehensive list can be found in the Supplementary material).

### Complete SARS-CoV-2 RBD or N immunoassay lyophilization in swab jackets and characterization

A 4× stock solution containing β10-labeled anti- SARS-CoV-2 RBD mAb (D002), β9-labeled anti- SARS-CoV-2 RBD mAb (D003), LgTrip, Fz, and excipients was prepared for the RBD model system. The same 4× stock was made for the N model systems but instead, with β10-labeled anti- SARS-CoV-2 N mAb 9,548 and β9-labeled anti- SARS-CoV-2 N mAb 9,547. We define the buffer (i.e., everything except antibody, LgTrip, and Fz) used to make this solution as “complete complex buffer.” Aliquots (400 μl) were prepared in disposable swab jackets for lyophilization. The swab jackets were loaded into the lyophilizer (Virtis Genesis 12EL) with shelves pre-set to 4°C. Product then underwent a freezing step with a shelf temperature of −50°C for 2 h. Upon evacuation of the system, the lyophilization process was performed between shelf temperatures of −25°C and 25°C and pressures of 75 and 200 mTorr. The ice sublimation phase lasted 5 h and the bound water desorption phase lasted 16 h. At the end of the lyophilization process, the swab jackets were covered with a temporary stopper under atmospheric conditions.

Immediately prior to use, the jackets were reconstituted with 400 μl of sample (100 μl) + PBSB mixture, shaken manually, and read on the handheld prototype luminometer every 15 min.

### Data analysis

Immunoassay dose response titrations were fitted to a 4-parameter logistic regression equation with 1/Y^2^ weighting function (GraphPad Prism 8). Limit of detection (LOD) was calculated as RLU_blank_ + 3 × SD_blank_. Signal over background (S/B) was calculated by dividing the raw Relative Light Unit signal for a given sample by the average of the background with buffer alone (*n* = 3). To quantify protein concentrations in samples, RLU values were interpolated on the standard curve.

## Results

### Solution-based homogeneous SARS-CoV-2 antigen immunoassay development

Prior to development of the proof-of-concept POCT, we first established solution-based, homogeneous assays targeting the SARS-CoV-2 nucleocapsid protein (N-antigen) or the SARS-CoV-2 RBD protein (RBD antigen). Both viral proteins are highly immunogenic with the nucleocapsid protein playing an essential role in replication and virion assembly while the RBD protein mediates viral binding of the host cell Angiotensin-converting enzyme 2 (ACE2) receptor (Lutomski et al., 2021). We have previously engineered the ternary Nluc reporter peptides by splitting Nluc, a 10-strand β-barrel luciferase, at the C-terminus at strands 9 and 10 resulting in two small peptides and a larger polypeptide, LgTrip (Hall et al., 2021). HiBiT, a high-affinity strand 10 peptide, has been identified as the preferred strand 10 peptide (β10) based on luminescent signal output in the ternary system and was carried through for these experiments. We further evolved the strand 9 (β9) derived peptide as well as LgTrip to overcome poor aqueous solubility and improve protein stability (Hall et al., 2021; Kincaid et al., 2022; Supplementary Figure 1). To create the chemical conjugation reporter tags, we relied on HaloTag-mediated chemical labeling of a pair of complementary antibodies that recognized non-overlapping epitopes of the target analyte (Nath et al., 2017).

**Figure 1:**
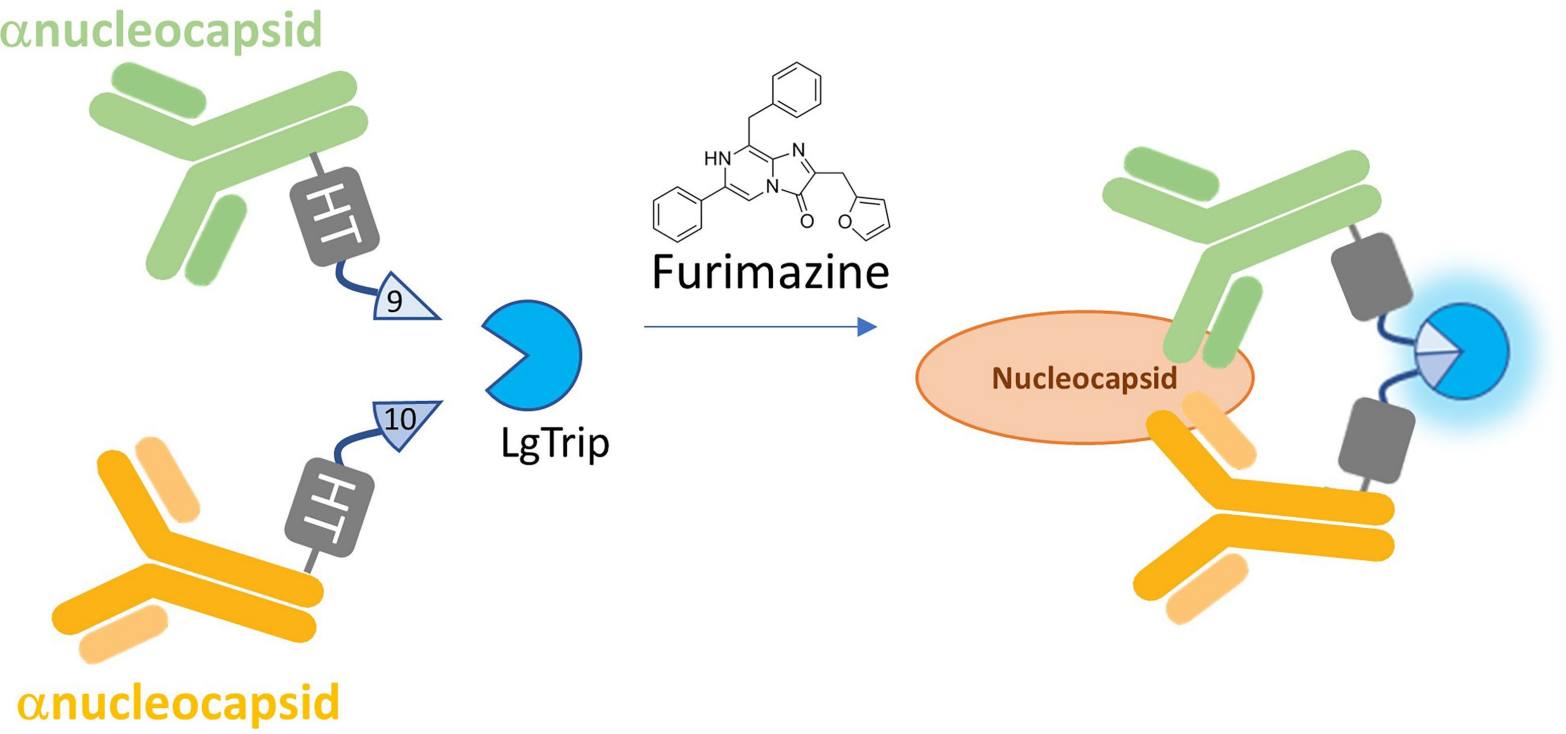
Schematic of the ternary Nluc reporter immunoassay. β9 and β10 peptides are chemically conjugated *via* HaloTag (HT) to a pair of antibodies that recognize separate epitopes on the target analyte. Binding to the analyte brings the antibodies and their respective peptide subunits into close proximity. With the addition of LgTrip and the furimazine substrate, complementation of the active ternary Nluc complex occurs, producing bioluminescence.

Commercially available complementary antibody pairs specific to each target protein from the Wuhan-Hu 1 strain were chemically conjugated with either HaloTag- β9 or HaloTag- β10 proteins. Binding to the target analyte brings both antibodies into proximity and allows the complementation of the active ternary Nluc complex with the addition of LgTrip and a bioluminescent signal can be measured in the presence of the Nluc substrate, Fz, as shown in the schematic in Figure 1. The design of the system required 4 antibody conjugates (antibody 1-HT-β9, antibody 1-HT-β10, antibody 2-HT-β9, and antibody 2-HT-β10) that were paired for matrix experiments (Supplementary Figure 2) for complete evaluation. Both combinations performed well with a protein standard; therefore, we selected the orientation that provided the highest signal-to-background (S/B) ratio. Next, we determined the optimal concentrations of the labeled antibodies for each target analyte immunoassay by conducting a matrix experiment using serial dilutions of each antibody under saturating conditions of the LgTrip and Fz substrate in the presence or absence of analyte (Supplementary Figure 2A). The S/B was calculated to identify the concentration of each antibody that produced the maximal sensitivity and dynamic range (Supplementary Figures 2B–E). Results for the N-antigen assay returned optimal concentrations of 30 ng/ml HT- β9-labeled anti-SARS-CoV-2 N mAb Cat. 9,547 and 60 ng/ml HT- β10 labeled anti-SARS-CoV-2 N mAb Cat. 9,548. The RBD assay final affinity reagent concentrations were 60 ng/ml HT- β9-labeled anti-SARS-CoV-2 spike RBD antibody D003 and 120 ng/ml HT- β10-labeled anti- SARS-CoV-2 spike RBD antibody D002 (Supplementary Figures 2B–G).

**Figure 2:**
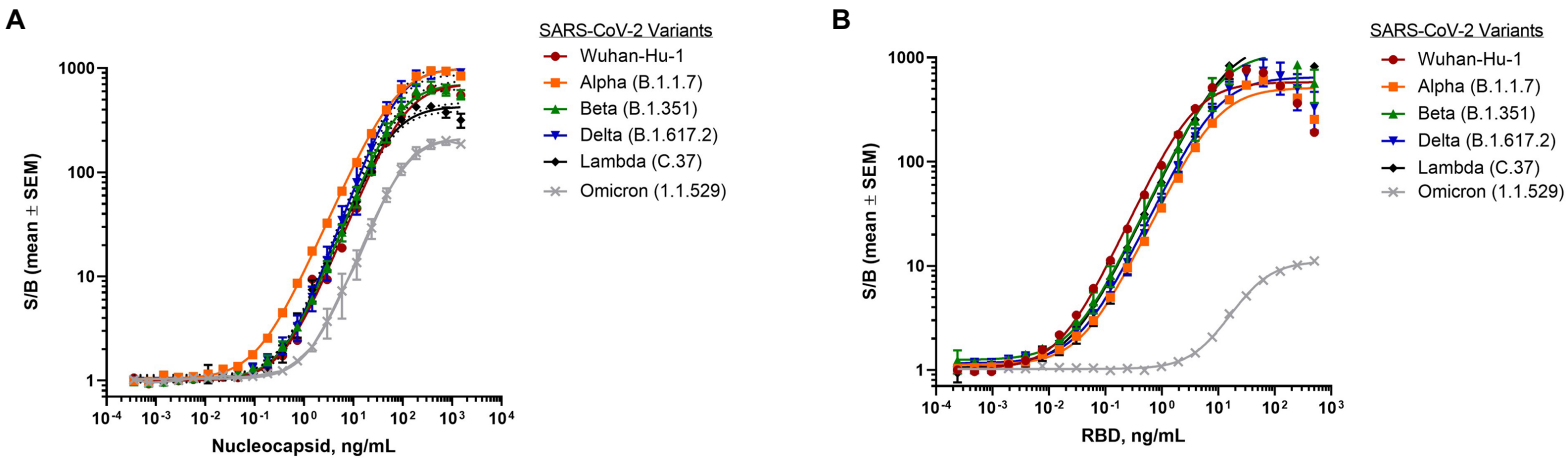
Detection of SARS-CoV-2 purified proteins from different variants. **(A)** Dose-response curve of purified nucleocapsid proteins from variants using nucleocapsid antigen assay. Final antibody concentrations were 30 ng/ml β9-labeled anti-SARS-CoV-2 N mAb Cat. 9,547 and 60 ng/ml β10-labeled anti-SARS-CoV-2 N mAb Cat. 9,548. **(B)** Dose-response curve of purified RBD proteins from variants using RBD antigen assay. Final antibody concentrations were 30 ng/ml β9-labeled anti-SARS-CoV-2 spike RBD antibody D003 and 60 ng/ml β10-labeled anti- SARS-CoV-2 spike RBD antibody D002. 1 μM LgTrip was used in all experiments. Reagents were incubated for 90 min prior to the addition of Fz substrate. Luminescence was measured and the signal over background (S/B) was calculated for each concentration of purified viral protein. Shown are means ± SEM (*n* = 3 independent experiments).

To establish assay performance, dose-response curves were generated using a simple, homogeneous workflow with viral proteins added directly to the plate, followed by a master mix including all assay reagents. This plate-based format has advantages of being fast, sensitive, and high-throughput, thereby potentially alleviating key issues routinely encountered with ELISAs in central laboratory settings (Supplementary Figure 3; Kozel and Burnham-Marusich, 2017; Sakamoto et al., 2018). Assay performance was established for both the N-antigen as well as the RBD antigen using recombinant proteins from various SARS-CoV-2 variants. The N-antigen assay displayed great sensitivity across all tested recombinant SARS N-protein samples from different SARS-CoV-2 variants, including Wuhan-Hu-1 (LOD = 0.108 ng/ml), Alpha (LOD = 0.019 ng/ml), Beta (LOD = 0.066 ng/ml), Lambda (LOD = 0.089 ng/ml), Delta (LOD = 0.050 ng/ml), and Omicron (LOD = 0.522 ng/ml) with a dynamic range spanning about four orders of magnitude (Figure 2A).

Similarly, the RBD antigen assay demonstrated great sensitivity across the majority of SARS-CoV recombinant RBD proteins resulting in LODs for Wuhan-Hu-1 (0.007 ng/ml), Alpha (0.009 ng/ml), Beta (0.004 ng/ml), Delta (0.006 ng/ml), and Lambda (0.002 ng/ml) with a dynamic range spanning three orders of magnitude (Figure 2B). Interestingly, the assay sensitivity was found to be significantly lower for the Omicron variant RBD protein (2.2 ng/ml) (Figure 2B), which carries 15 mutations known to impact antibody binding (Wu et al., 2022).

In addition, recombinant N and RBD antigens from different coronaviruses including SARS-CoV, Middle East Respiratory Syndrome coronavirus (MERS-CoV), and a variety of low pathogenicity human coronaviruses, were tested to determine assay cross-reactivity. Both antigens from SARS-CoV were recognized in this format with high sensitivity which is not surprising considering the high degree of homology found between SARS-CoV and the Wuhan-Hu-1 strain of SARS CoV-2 (Grifoni et al., 2020). Importantly, the N-antigen assay did not cross-react with any of the recombinant proteins from low pathogenicity coronaviruses or MERS-CoV demonstrating excellent specificity (Supplementary Figure 4A) across pathogenic coronaviruses. Surprisingly, we found substantial cross-reactivity in the RBD antigen assay for coronavirus strain HCoV-NL63 spike protein that might be attributed to epitope sequence homology (Supplementary Figure 4B; Simula et al., 2020). This finding in combination with lower observed sensitivity against the Omicron variant suggests potential liabilities with utilizing the RBD antigen for specific detection of SARS-CoV-2 in a POCT.

Taking into consideration that speed is a key requirement for POCT to accommodate rapid sample-to-answer workflows, we next examined the kinetics of the signal formation for both antigen assays. Signal intensity and kinetics are driven by the complementation and maturation of the ternary Nluc components into a functional luciferase complex, affinity and avidity of the antibodies, and the concentration of the analyte. When all the assay components were added at *t = 0*, we observed that the development of the luminescence signal was both time- and analyte concentration-dependent as expected (Supplementary Figures 5A–D). Both assays exhibited greater sensitivity over time but did not vary in terms of signal amplitude. Importantly, kinetic analysis revealed that the analyte-specific signal is sustained for an extended period of time without a rise in background signal and concomitant loss of sensitivity. Analysis of assay performance over time indicates stability for at least 90 min without discernible loss in sensitivity (Supplementary Figures 6A,B). This signal stability highlights the technology’s robustness which permits reliable sample analyses across a broad time window without increased occurrence of false results. This marks a significant improvement over many current standard ELISA and point-of-care assay formats which often require narrow time windows for obtaining reliable results.

### Assay performance using heat-inactivated virus and clinical nasopharyngeal samples

To establish assay performance against clinically relevant viral samples, we next analyzed the ability of the assay to detect heat-inactivated virus. Surprisingly, when tested with heat-inactivated viral SARS-CoV-2 variants Wuhan-Hu 1, Alpha (B.1.1.7), and Beta (B.1.351), the RBD antigen assay failed to produce a signal despite increasing amounts of virus (Supplementary Figure 7A). To determine if the poor performance could be attributed to heat-induced damage of the target epitope, the RBD antigen assay was also tested using non-heat treated residual clinical nasopharyngeal (NP) samples obtained from our clinical collaborator at the University of Wisconsin Hospital and Clinics. Individual NP samples that had previously been identified by PCR as negative (*n* = 10) or positive (*n* = 10) were tested in the RBD antigen assay and no significant difference in signal generation was found (*p* = 0.17; Supplementary Figure 7B) between positive and negative samples. Due to these results, the SARS-CoV-2 RBD antigen assay was abandoned for further assay development.

**Figure 3:**
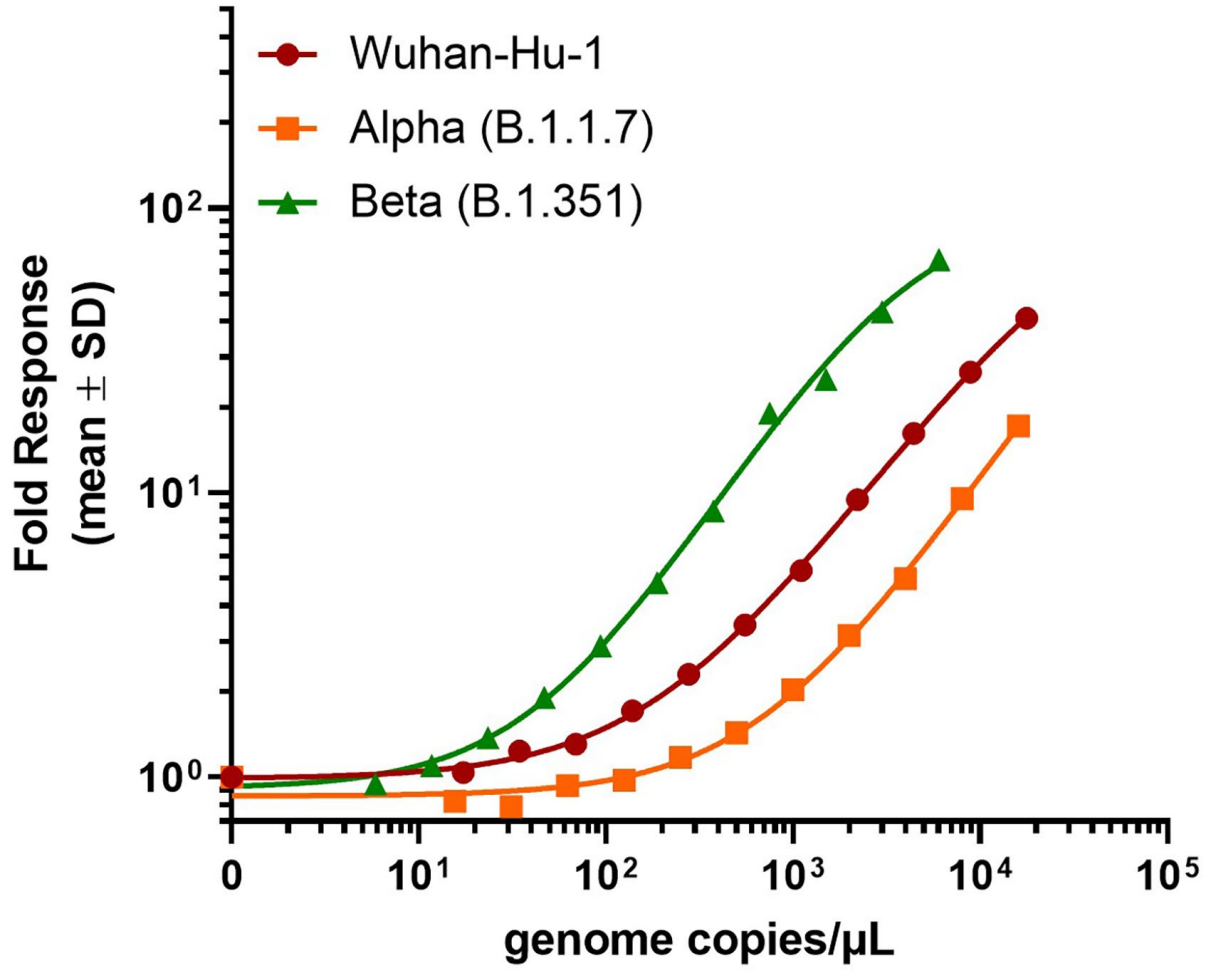
Detection of SARS-CoV-2 heat-inactivated viruses with N-antigen assay. 30 ng/ml β9-labeled anti-SARS-CoV-2 N mAb Cat. 9,547, 60 ng/ml β10-labeled anti-SARS-CoV-2 N mAb Cat. 9,548, and 1 μM LgTrip were incubated in the presence of sample for 90 min prior to the addition of Fz substrate and the measurement of luminescence. Signal over background (S/B) was calculated for each concentration of heat-inactivated virus. Shown mean ± SD of a representative experiment.

In contrast, the SARS-CoV-2 N-antigen assay displayed a dose-dependent response across all heat-inactivated SARS-CoV-2 variants tested (Figure 3). Interestingly, the assay demonstrated a decrease in sensitivity for the Alpha (B.1.1.7) variant (LOD = 242 genome copies/μl) and an increase in sensitivity for the Beta (B.1.351) variant (LOD = 26 genome copies/μl) relative to the Wuhan-Hu-1 variant (LOD = 74 genome copies/μl), which was used to generate N-antigen specific antibodies. These results differ from what was previously observed when using purified recombinant N-antigen (Figure 2A) and could indicate differences in the epitope presentation in the naturally occurring virus versus recombinantly expressed viral proteins. The results found for the Wuhan-Hu-1 variant were expected given that the α-nucleocapsid antibodies for the assay were developed against this variant during the original wave of the pandemic. It was previously shown that post-translational modifications of RBD varied significantly between different SARS-CoV-2 variants. We hypothesized that the poor performance could be attributed to a variety of factors, including lack of accessibility due to glycosylation of the spike protein or low abundance of the target epitope in samples with a whole virus (Ke et al., 2020; Shajahan et al., 2020). The nucleocapsid protein of the Alpha (B.1.1.7) variant has been shown to be heavily phosphorylated relative to the original Wuhan-Hu-1 strain, while the Beta (B.1.351) has fewer post-translational modifications (PTMs; Supekar et al., 2020; Mohammad et al., 2021). We speculate that differences in PTM composition and abundance could lead to altered epitope presentation and therefore impact the binding of antibodies to the viral nucleocapsid protein which would explain the observed differences in sensitivity.

**Figure 4:**
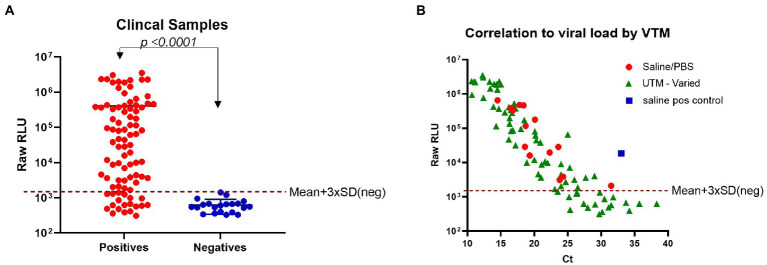
Detection of SARS-CoV-2 N-antigen in clinical samples. **(A)** Clinical samples that had previously been identified by PCR as negative (*n* = 21) or positive (*n* = 89) were individually tested in the N-antigen assay. **(B)** N-antigen assay raw RLU results were correlated to viral load (Ct) by VTM for all PCR positive samples (*n* = 89). In both panels a and b, 30 ng/ml β9-labeled anti-SARS-CoV-2 N mAb Cat. 9,547, 60 ng/ml β10-labeled anti-SARS-CoV-2 N mAb Cat. 9,548, and 1 μM LgTrip were incubated in the presence of sample for 90 min prior to the addition of Fz substrate and the measurement of luminescence. A red, dotted line has been added to each panel to represent a cut-off based on Mean + 3 × SD (neg). Statistical analyses determined by using an unpaired, non-parametric Mann–Whitney t-test.

To demonstrate the clinical utility of the N-antigen assay, residual clinical NP samples that were previously PCR-confirmed negative (*n* = 21) or positive (*n* = 89) were analyzed using the plate-based format of the assay to allow for a high-throughput approach given the large number of samples being tested (Supplementary Figure 3). The N-antigen assay showed great specificity with all negatives grouping tightly at low RLU values (Figure 4A). The positive cohort had a dynamic range covering four log orders of signal magnitude, highlighting the benefit of using a bioluminescence-based reporter, which minimizes the need to dilute samples with high analyte concentrations while preserving sensitivity due to its inherently low background (Figure 4A). The results demonstrated a clear and significant delineation between samples (*p* < 0.0001; Figure 4A). In a further effort to categorize samples as positive or negative in the immunoassay in a standardized fashion, we established a cut-off value using the mean + 3 × SD of RLU values obtained from all PCR negative samples. Based on this cut-off, there was some discordance in outcome between the PCR results and the N-antigen assay resulting in 19/89 samples considered as false-negatives (Table 1). As these samples were residual clinical samples, we acknowledge the liabilities with the sample set including poor antigen stability due to prolonged and improper sample storage, variety of viral transport media (VTM) that could affect protein stability, as well as samples containing very low viral loads which could all contribute to the false-negatives reported here.

**Table 1:** Overall assay performance.

	RT-PCR results			
**N-antigen test results**		**+**	**−**	
	**+**	70	0	70
	**−**	19	21	38
		89	21	108

In order to determine if low viral load could explain the discordance between PCR and antigen assay, we investigated the relationship between reported PCR cycle threshold (Ct) values and signal in the antigen assay. We compared the RLU values obtained with the developed immunoassay to the reported Ct values to determine if the false-negatives contained a low viral load. Sample viral load is inversely related to the Ct value and an increase of 3.3 in Ct value corresponds to a 10-fold reduction in viral RNA molecules (Al Bayat et al., 2021). We found good correlation between Ct values and RLU values produced by the N-antigen assay in samples with high viral load (Figure 4B). Previously, a Ct value ≤30 has been identified as a cut-off for viral infectivity with a markedly lower risk of secondary transmission to close contacts (Al Bayat et al., 2021). These false-positives have delayed hospitals in discharging non-infectious patients and have emphasized the need for reliable, accurate antigen tests to combat the pandemic (Braunstein et al., 2021). Samples with a Ct value ≤30 were detected with 69/79 concordance while samples with a Ct ≤ 25 had an improved concordance finding 64/65 in agreement. One factor that might have impacted N-antigen detection in PCR positive samples is the use of VTM for collection and storage of most tested clinical samples. VTM reagents include chemical additives typically optimized for downstream molecular testing and have been shown to denature proteins thus affecting performance in antigen detection assays. To illustrate the potential impact of VTM on the N-antigen assay performance, we tested the compatibility of a number of commonly used commercially available VTMs with the N-antigen assay. The experiment was conducted using a fixed concentration of purified SARS-CoV-2 nucleocapsid protein from the Wuhan-Hu-1 strain in the presence of a serial dilution of each VTM (Supplementary Figure 8). The data indicate that the choice of VTM has a significant impact on assay performance and should be therefore considered as a critical reagent. Certain VTMs, including PrimeStore® MTM and Hologic Aptima® were found to be completely incompatible with the assay even at high dilutions of the VTM. Other commercial VTMs were found to lead to various degrees of signal loss compared to PBS and saline in a concentration-dependent manner. This experiment demonstrates that the choice of sample medium is a critical factor for assay performance.

### Point-of-care assay development

**Figure 5:**
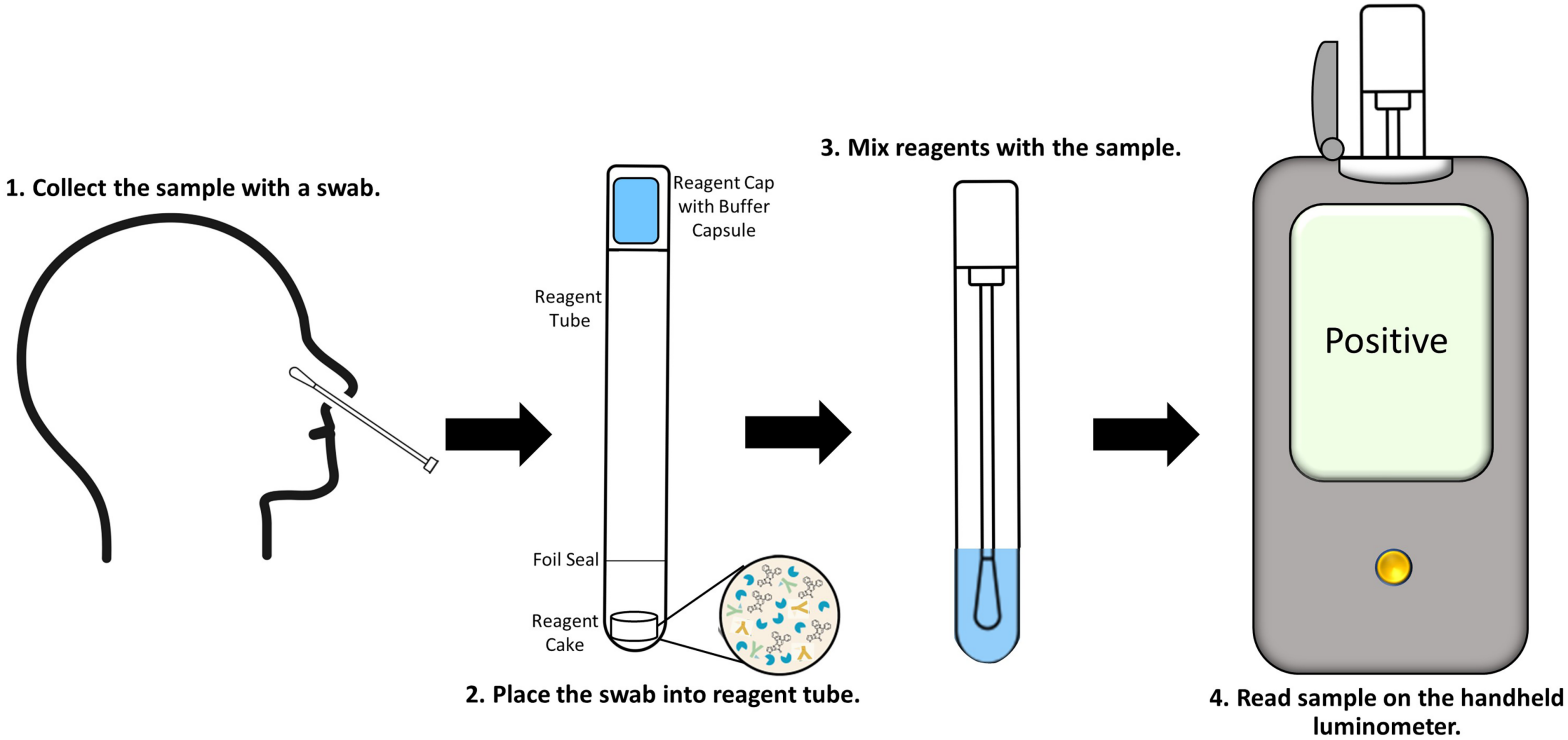
Proposed workflow for the point-of-care SARS-CoV-2 antigen immunoassay utilizing the ternary Nluc reporter system. Nasal swabs are collected (1) and placed into the reagent tube, breaking through the foil seal to expose the reagent cake (2). The cap is placed and locked onto the tube containing the sample. The buffer capsule in the cap is then cracked and the tube is shaken to reconstitute the cake and mix the assay reagents with the sample (3). The tube is then inserted into the handheld luminometer and read (4).

**Figure 6:**
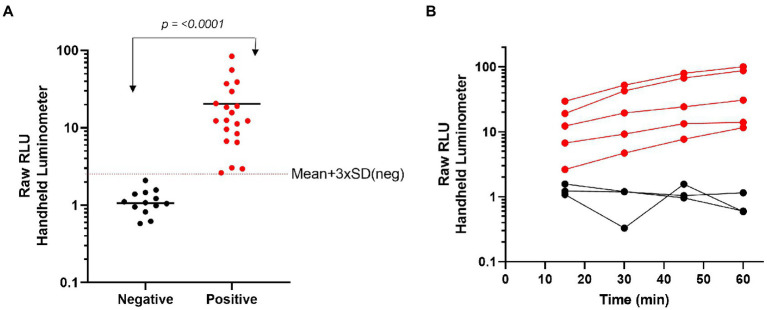
Characterization of a single-reagent SARS-CoV-2 antigen assay for point-of –care testing with nasal clinical samples. **(A)** Clinical samples that had previously been identified by PCR as negative (*n* = 13) or positive (*n* = 20) were added directly to the lyophilized N-antigen assay then read with a handheld luminometer after 15 min. **(B)** A subset of clinical samples were added to the lyophilized N-antigen assay and measured at 15 min intervals over a 1 h time period. Statistical analyses performed by using unpaired, non-parametric Mann–Whitney *t*-test.

We have previously demonstrated that through formulation and lyophilization, we can develop assays using a stable, all-in-one reagent that can be reconstituted in aqueous buffers and added directly to sample in standard microtiter plates (Hall et al., 2021). To further explore the potential use of this technology in a POCT format, we utilized the same formulation and lyophilization approach to create a prototype POCT with the assay reagent directly placed into a swab jacket as outlined in the schematic depicted in Figure 5. In this format individually collected samples are added directly to a swab jacket containing the all-in-one lyophilized N-antigen assay reagent and reconstitution buffer, briefly incubated, and then analyzed using a basic handheld luminometer. Using this approach, we generated a titration of recombinant N-antigen to establish a standard curve on the handheld device which showed a good correlation (*R*^2^ = 0.9676) with standard curve values measured on a plate reader (Supplementary Figure 9). Following this proof-of-concept experiment, we next examined the performance of the N-antigen immunoassay in a true POCT format. To this end, all components of the N-antigen immunoassay (peptide-labeled antibodies, LgTrip, and furimazine substrate) were directly lyophilized into a nasal swab jacket to mimic a homogeneous, rapid POCT format for the detection of SARS-CoV-2 antigen in clinical samples. Samples previously determined as positive (*n = 20*) or negative (*n = 13*) by both PCR and standard immunoassay were added directly to the swab jacket along with PBS reconstitution buffer (in a 100 μl:300 μl ratio) and analyzed using the handheld luminometer following a 15-min incubation period. The results demonstrated a clear and significant delineation between PCR-confirmed positive and negative samples (*p* < 0.0001; Figure 6A). For analysis, we utilized a cut-off value set at the mean + 3 × SD of all negative samples which returned a 100% specificity for the samples tested (Figure 6A). The positive samples displayed results spanning two orders of magnitude in dynamic range and showed a good correlation between the handheld and GloMax plate reader (Supplementary Figure 10). Assay development time is a critical parameter to be considered in POCT development. To understand the kinetics of the signal generation, a subset of samples was measured repeatedly in 15 min intervals over the course of 1 h (Figure 6B). The results of the kinetic read demonstrate the beneficial effects provided by the sustained bioluminescence glow reaction. The negative samples showed no increase in signal which minimizes the danger of false-positives due to overdevelopment over time (Figure 6B) which is a common problem in colorimetric formats (Terato et al., 2014; Mouliou and Gourgoulianis, 2021). Of equal importance is signal stability for preventing positive samples returning false-negative results over time due to loss of signal. We observed that positive samples provided a high RLU value that increases over time. The improved signal separation seen with a longer reading window could potentially improve assay sensitivity, if needed. Flexible assay incubation times allow users to batch analyze samples and streamline workflows in a point-of-care environment without compromising on assay accuracy. We also used the kinetic workflow to highlight the potential for sample quantitation by calculating the concentration of nucleocapsid protein in each positive sample at 30 or 60 min based on the Wuhan-Hu-1 standard curve generated with the handheld device (Supplementary Figure 11). The sample quantitation covered a broad range at both time points but showed improved separation between samples after 60 min.

**Figure 7:**
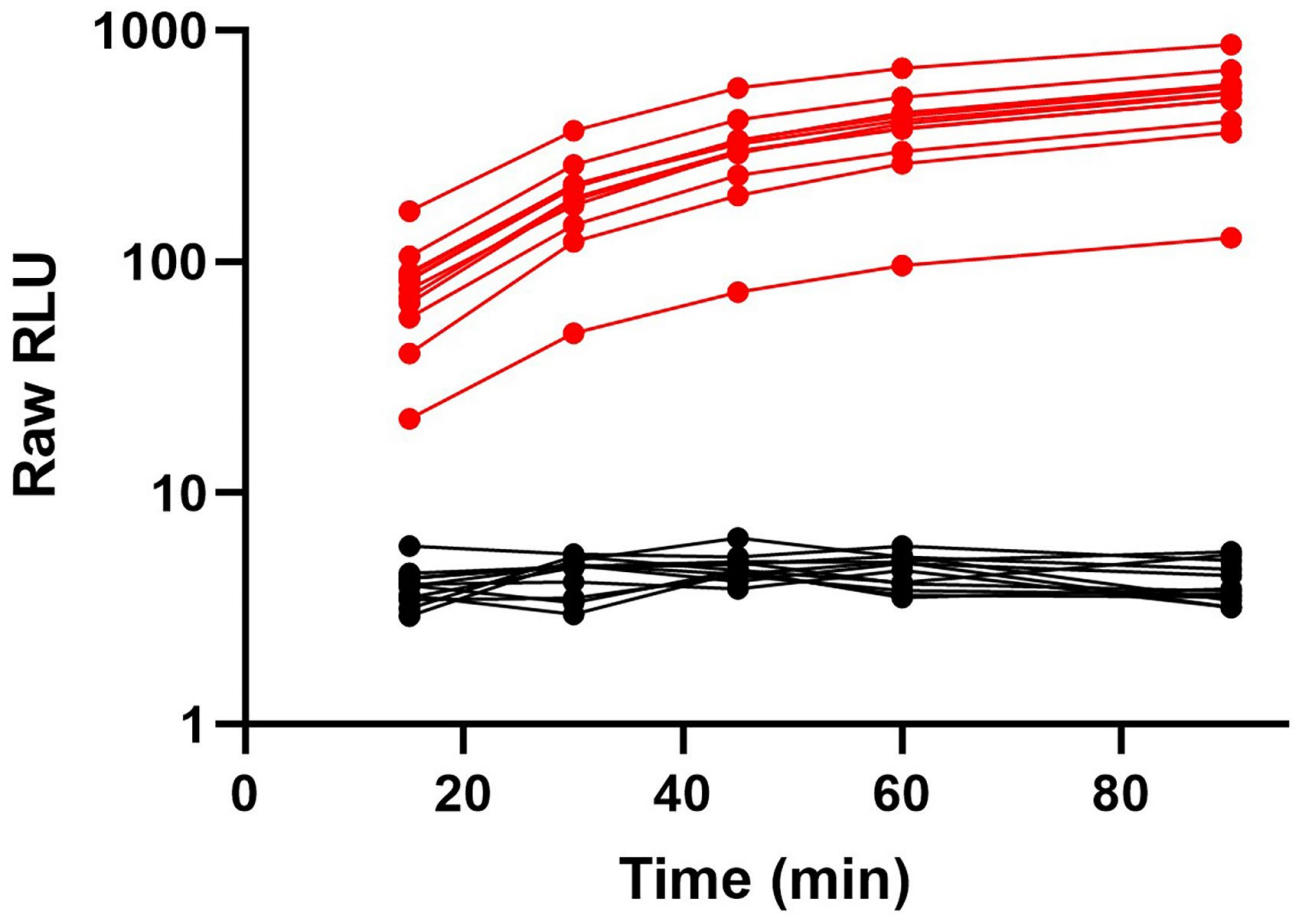
Ternary Nluc-based assay for SARS-CoV-2 antibody response in serological samples. Serum samples from convalescent SARS-CoV-2 patients (*n* = 13) in red or pre-pandemic patients (*n* = 12) in black were individually tested with the lyophilized, all-in-one ternary Nluc serology assay. Samples were measured at 15, 30, 45, 60, and 90-min intervals with a handheld luminometer.

To further showcase the flexibility and performance of the ternary Nluc technology POCT platform, we developed an assay to detect SARS-CoV-2 antibodies in human plasma and serum samples using RBD-Trip peptide genetic fusions. Serological tests can provide information about serostatus from previous infections as well as post-vaccination and are frequently used for public health decision-making (West et al., 2021). For this model, we generated RBD- β9 and RBD- β10 genetic fusions which negates the need for chemical labeling. A combination of these fusions was used to detect anti-SARS-CoV-2 antibodies present in serum or plasma. Because of the bivalent nature of antibodies incubating positive serum with an equimolar mixture of both RBD genetic fusions will result in half of the RBD-specific antibodies binding simultaneous to one molecule of RBD- β9 and RBD- β10 each, thus bringing the β9 and β10 into close proximity. Upon addition of the detection reagent containing the LgTrip and furimazine substrate, reconstitution of an active enzyme complex will result in a luminescent signal that is proportional to the level of the anti-SARS-CoV-2 antibody present in the sample. A schematic of this assay can be found in Supplementary Figure 12. The ability to use this same chemistry in combination with genetic fusions offers yet another aspect of flexibility to the ternary Nluc assay design. Genetic fusions add a single tag to the detection proteins at a defined position, which simplifies manufacturing by removing the need for a chemical purification and decreases lot-to-lot variability. To create a POCT prototype for SARS-CoV-2 serology analyses, all components including furimazine substrate were lyophilized directly into swab jackets for an add-sample-and-read assay format. Clinical samples were collected from convalescent patients (*n* = 13) who had experienced a natural SARS-CoV-2 infection prior to the administration of vaccination or negative samples commercially sourced pre-pandemic (*n* = 12). The reagent cake was resuspended in PBS buffer and added directly to samples in a swab tube. Luminescence was read in the handheld device at 15, 30, 45, 60, and 90 min (Figure 7). Within this limited study, the assay showed great specificity using a cut-off derived from the mean + 3 × SD of all negative samples. The positive samples showed excellent sensitivity with a dynamic range spanning two orders of magnitude with no apparent interference from the serum sample-matrix. The assay also displayed sustained signal kinetics over time allowing for flexible read times but importantly preventing false-negatives or false-positives that can be associated with changes in signal over time as frequently observed with standard colorimetric or fluorescent reporter systems used for POCT (Mouliou and Gourgoulianis, 2021).

## Discussion

There is a continued need to expand the ability to detect clinically relevant analytes at the site of the patient using POCT. This need is particularly relevant for monitoring and controlling emerging infectious diseases such as the ongoing SARS-CoV-2 pandemic. We have previously introduced the ternary split-Nluc platform as a bioluminescence-based homogeneous immunoassay that can be readily adapted for a range of model systems and herein adapt this technology for POCT (Hall et al., 2021). The platform is highly flexible since two of the three reporter fragments are small peptides and can be used to chemically label any amenable molecules. The other components, LgTrip and formulated furimazine, have been optimized to allow for all-in-one lyophilized assays suitable for ambient temperature storage in resource-limited settings. The ternary system offers major advantages over other systems in that the affinity reagent concentrations are separate from the assay signal generation, broadening the dynamic range of the assay at the upper end of analyte concentrations. During assay development, the concentration of peptide-labeled antibodies can be independently adjusted with saturating concentrations of the LgTrip detection reagent and furimazine substrate to obtain the optimal assay performance.

In this study, we introduce a proof-of-concept for POCT by adapting a flexible, all-in-one technology for a SARS-CoV-2 model system. Initially, we pursued two potential SARS-CoV-2 antigens, the nucleocapsid protein (N) and the RBD protein with the ternary Nluc platform. Each immunoassay was initially optimized in a homogeneous plate-based format with purified recombinant proteins prior to transitioning to a point-of-care format. While the dynamic range for the assays spanned multiple orders of magnitude, the assay signal for both antigens decreased at extremely high concentrations of proteins, a phenomenon common to all homogeneous immunoassays known as the “Hook effect”(Ross et al., 2020). Although the loss in signal never resulted in a false-negative reading, absolute quantitation of the analyte once outside of the linear range would not be possible. This effect could be addressed by using a higher concentration of antibodies to capture the higher end of analyte concentrations if quantitation was needed.

Surprisingly, while the RBD assay performed well with purified proteins, it failed to detect antigen in either the heat-inactivated virus or clinical NP samples. Currently, all commercially available antigen assays use nucleocapsid protein as the target analyte, indicating others have noted limitations with using RBD as a target analyte (Liu et al., 2021). Given the poor performance with clinical samples, we abandoned RBD as a target and advanced with the N-antigen assay.

Overall, the plate-based N-antigen immunoassay was two orders of magnitude more sensitive than reported values for all commercially available high-throughput and RATs (Scohy et al., 2020; Eshghifar et al., 2021). Currently available RATs have been plagued by low sensitivity in clinical practice with LODs well below manufacturer-reported values, especially in asymptomatic cases (Kahn et al., 2021). Such a drastic improvement in sensitivity could be crucial for combating the rise of new variants and further spread of SARS-CoV-2, especially with the growing reliance on direct-to-consumer RATs by the public.

Given the superior performance of the N-antigen assay with the model systems, we moved forward with a small set of residual patient samples in an add-and-read approach. We recognize development of this prototype would require a much larger sample cohort in order to establish a reliable threshold value for distinguishing positive from negative samples. However, using the limited number of clinical samples available allowed us to demonstrate a prototype test that showed overall good discrimination between positive and negative samples.

Several PCR-confirmed positive samples (19/89) returned a potential negative with the N-antigen assay, indicating some discrepancy between the two assays. We hypothesized that there were liabilities with the limited sample set including storage conditions such as multiple freeze/thaw cycles, temperature, and VTM. Potential VTM interference with RAT performance has been well documented, specifically in causing false results (Cubas-Atienzar et al., 2021; Patriquin et al., 2021; Zhou et al., 2021). We obtained commercial VTMs and verified their compatibility with the N-antigen assay. Based on this work, we moved forward with PBS for the assay media, allowing for direct sample addition to the assay without additional handling or dilution of the target analyte. This add-and-read approach streamlined the overall workflow and pushed the assay further into the POCT space.

We have previously shown that the ternary Nluc bulk reagents can be reformulated from a solution-based format to a lyophilized, all-in-one format to address different sample types and application needs (Hall et al., 2021). Taking the next step, the N-antigen assay (including Fz substrate) was converted into a POCT prototype format, with all the assay components from the solution-based work lyophilized into a cake within a nasal swab tube. To demonstrate utility of this format in a realistic POCT setup we used a handheld luminometer for data collection. Within 15 min, our N-antigen POCT was able to distinguish between SARS-CoV-2 positive and negative samples with a high degree of confidence. This underscores the potential of this technology for use in POCT applications.

Using the same format, we measured clinical samples over the course of a 1 h period to demonstrate that the assay results were stable over extended periods of time. In this way, the ternary Nluc system provides a major benefit over other reporter technologies that suffer from overdevelopment of the reporter or waning signal over time, thereby potentially impacting validity of assay results over time. The extended read window for the assay could allow assay users and technicians to test samples more efficiently in large batches without having to worry about false results.

Given the broad dynamic signal range observed with positive clinical samples, we also attempted to quantify the amount of nucleocapsid protein, or the amount of virus based on our previously established standard curve (Supplementary Figure 9). The sheer number of SARS-CoV-2 variants and the numerous mutations within those variants confounds antigen quantitation, especially in clinical samples. The rapid evolution of SARS-CoV-2 represents a rather unique situation, which implies that quantifying other clinically relevant analytes (e.g., troponin, CRP, etc.) with the Nluc technology would likely be more straightforward and could offer major benefits at the point-of-care.

Though PCR-based techniques have served as the primary diagnostic for SARS-CoV-2 infections, global testing capacities have been strained, which emphasizes an urgent need for antigen tests that meet the set of criteria published by the World Health Organization for POC diagnostics known by the acronym ASSURED (affordable, sensitive, specific, user-friendly, rapid/robust, equipment-free/minimal equipment, and deliverable to end-users) for better disease control (Land et al., 2019). The modular nature of the ternary Nluc technology allowed us to rapidly design and develop a serological assay detecting immune responses against SARS-CoV-2. Using this model system, we showcased another accommodating assay feature of the ternary, split-Nluc platform, namely the ability to append β9 and β10 peptides as genetic fusions rather than the chemical labeling approach used in the antigen assay. This and a recently published study have focused on transitioning the ternary Nluc technology from HaloTag-based labeling approach to direct peptide labeling and genetic fusions to enable flexible labeling options for different targets and model systems(Kincaid et al., 2022). The direct labeling and genetic fusion methods use smaller peptide tags that are bioinert and can be easily appended anywhere on targets of interest. Genetic fusions add an additional benefit in being able to control the number of labels added to the target. All labeling options rely on the same chemistry to generate bioluminescence thereby allowing the same flexibility across all assay formats including solution-based and lyophilized all-in-one.

The same chemistries used in the ternary Nluc solution-based assays translate directly into lyophilized formats while maintaining performance, which offers unique assay flexibility compared to other available technologies. Herein, we were able to lyophilize the reporter system and formulated substrate into a shelf-stable, homogeneous, add-and-read analyte detection reagent cake for point-of-care applications using a simple handheld luminometer. POCT is valuable in providing rapid results outside of a typical clinical laboratory setting with simplified workflows, but often can suffer in sensitivity, sample-matrix interference, and is often limited to qualitative results. As demonstrated in this work, the ternary Nluc technology overcomes current limitations in the point-of-care space, enabling a rapid, sensitive, robust platform for a variety of targets and model systems.

## Data availability statement

The original contributions presented in the study are included in the article/Supplementary material, further inquiries can be directed to the corresponding author.

## Author contributions

ET and VR purified proteins, performed all immunoassays and calibrations, conducted data analysis, and wrote the manuscript. WR and MA provided nasopharyngeal swab samples and associated clinical data. VK, MH, and LE designed and evolved the stabilized version of the ternary Nluc system. KZ constructed plasmids for all protein components. RH and C-CH expressed, purified, and characterized proteins described in the manuscript including LgTrip and genetic fusion proteins. SF conducted all lyophilization runs and optimized lyophilization conditions for all assays described in the manuscript. TM and MD performed data analysis, conceptualized the work, and designed the overall project scope. All authors contributed to the article and approved the submitted version.

## Conflict of interest

ET, VR, VK, RH, MH, LE, KZ, SF, C-CH, TM, and MD were employed by the company Promega Corporation.

The remaining authors declare that the research was conducted in the absence of any commercial or financial relationships that could be construed as a potential conflict of interest.

## Publisher’s note

All claims expressed in this article are solely those of the authors and do not necessarily represent those of their affiliated organizations, or those of the publisher, the editors and the reviewers. Any product that may be evaluated in this article, or claim that may be made by its manufacturer, is not guaranteed or endorsed by the publisher.
