# Supplementary material for: Development of a rapid, simple, and sensitive point-of-care technology platform utilizing ternary NanoLuc
Source: Front Microbiol. 2022 Oct 26;13:970233. doi: 10.3389/fmicb.2022.970233 (PMC9643700; doi:10.3389/fmicb.2022.970233)
Supplement: Supplementary file 1 [file Presentation_1.pdf]

Supplementary Material

1 Supplementary Methods

1.1 LgTrip Purification.

For purification of LgTrip, a plasmid expressing LgTrip fused to an N-terminal 6X HisTag with a T7 promoter was transformed into *E. coli* KRX cells (Promega). The transformed cells were cultured overnight in LB broth containing 0.025 mg/mL (w/v) kanamycin at 37°C. A 1:100 dilution of the overnight culture was transferred into 500 mL of fresh LB in the presence of 0.1% (w/v) rhamnose, 0.15% (w/v) glucose, 0.025 mg/mL (w/v) kanamycin and grown for 16-24 hours at 25°C. The cells were pelleted by centrifugation at 5000X g for 15minutes, re-suspended in a lysis buffer containing 50 mM Tris, pH 7.5, 150 mM NaCl, 1X Protease Inhibitor Cocktail (Promega), and 0.2 mg/ml (w/v) of lysozyme and then disrupted by 3 cycles of freeze/thaw. The disrupted cells were incubated on ice for 30–45 minutes with 0.5 ml RQ1 DNAase I and 10X RQ1 DNase I buffer (Promega). The supernatant was obtained by centrifugation at 10,000 x g for 30 minutes at 4 °C. The supernatant was made to be 20 mM Imidazole and 350 mM NaCl and then was applied to a 5 ml HisTrap FF (Cytiva) pre-equilibrated with Buffer A (50 mM Tris, pH 8.0, 500 mM NaCl, 20 mM Imidazole, 0.005% IGEPAL and 10% glycerol). 6X His-LgTrip was eluted with Buffer B (50 mM Tris, pH 8.0, 500 mM NaCl, 500 mM Imidazole,0.005% IGEPAL and 10% glycerol) using a 10-column volume (CV) gradient. The fractions were pooled and dialyzed (Slide-A-Lyzer Dialysis Cassettes 3,500 MWCO, ThermoFisher Scientific) against a buffer containing 35 mM Tris, pH 7.5, 350 mM NaCl, 0.005% IGEPAL and 50% glycerol when the protein was used for solution-based assays. When the LgTrip was used in the lyophilized stock solutions the protein was dialyzed against a buffer containing 25 mM Tris, pH 7.5, 350 mM NaCl and 10% sucrose.

1.2 Purification of HaloTag® Fusions to Peptide 840 and VS-HiBiT.

Colonies of KRX *E. coli* cells (Promega) transformed with HT- β9 (peptide 840) or HT- β10 (Vs-HiBiT) plasmids were inoculated into starter cultures containing LB broth + antibiotic and grown for 18 h at 37 °C. After, 1:100 dilutions of the overnight cultures were made with 500 mL of LB + 0.1% Rhamnose + 0.15% Glucose + antibiotic and grown for 20 h at 25 °C. The overexpressed cells were pelleted and re-suspended in a lysis buffer containing Phosphate-buffered saline (PBS) with 0.2 mg/mL Lysozyme (Sigma) and 1X Protease Inhibitor Cocktail (Promega). A lysate was prepared by subjecting the sample to three freeze/thaw cycles (between -80°C and room temperature). After the final thaw, 0.001 U/mL RNase-free RQ1 DNase (Promega) was added to the sample and incubated on ice for 30 min. The lysate was centrifuged, and the supernatant was collected and HaloTag-VS-HiBiT was purified using batch methodology with Ni Sepharose 6Fast Flow (GE) using the manufacturer's recommended protocol. HaloTag- β9(peptide 840) was purified using FPLC HisTrap FF 5 ml columns (Cytiva). Protein was eluted using a gradient elution up to 500 mM imidazole, dialyzed in PBS, and characterized to be >95% pure using SDS-PAGE. Proteins were stored in PBS + 50% glycerol at -20°C.

1.3 Acquiring Coronavirus Proteins and Heat-Inactivated Virus Strains.

The following recombinant, purified SARS-CoV-2 or coronavirus Spike/RBD proteins were purchased for use in this work: MERS-CoV Spike/RBD Protein fragment Cat. 40071-V08B1 (Sino), Human coronavirus (HCoV-229E) Spike/S1 Protein Cat. 40601-V08H (Sino), Human coronavirus HKU1 (isolate N1) (HCoV-HKU1) Spike Cat. 40021-V08H (Sino), Human coronavirus (HCoV-OC43) Spike S1 Protein Cat. 40607-V08H1 (Sino), Human coronavirus (HCoV-NL63) Spike/S1 Protein Cat. 40600-V08H (Sino), SARS-CoV Spike/RBD Protein (RBD, His Tag) Cat. 40150-V08B2 (Sino), and SARS-CoV-2 Spike S1+S2 (L5F, T95I, D253G, S477N, E484K, D614G, A701V) Protein (ECD, His Tag) Cat. 40589-V08B21 (Sino).

The following recombinant, purified SARS-CoV-2 or coronavirus nucleocapsid proteins were purchased for use in this work: SARS-CoV-2 Nucleocapsid (D63G, R203M, D377Y) Protein (His Tag) Cat. 40588-V07E29 (Sino), SARS-CoV-2 Nucleocapsid (P13L, R203K, G204R, G214C) Protein (His Tag) Cat. 40588-V07E31 (Sino), SARS-CoV-2 (2019-nCoV) Nucleocapsid (T205I) Protein (His Tag) Cat. 40588-V07E9 (Sino), SARS-CoV-2 Nucleocapsid (P199L, M234I) Protein (His Tag) Cat. 40588-V07E18 (Sino), and SARS-CoV-2 B.1.1.529 (Omicron) Nucleocapsid Protein (His Tag) Cat. 40588-V07E34 (Sino).

The following heat-inactivated virus strains were purchased for use in this work: Heat-inactivated SARS-CoV-2 Cat. VR-1986HK™ (ATCC), Heat-inactivated SARS-CoV-2 variant B.1.1.7 Cat. VR-3326HK™ (ATCC), and Heat-inactivated SARS-CoV-2 variant B.1.351 (Beta) Cat. VR-3327HK™ (ATCC).

1.4 Calibrating the Luminescent Signal of the GloMax to the Handheld Luminometer.

A 4X solution containing recombinant Wuhan-Hu-1 nucleocapsid protein was prepared for a dose-response curve and 150 µL was added to the jackets containing the master mix. A 30-fold dilution of Fz (Nano-Glo Live Cell Substrate; Promega N205) in PBSB was prepared and added at 300 µL/swab jacket for a final volume of 600 µL/swab jacket. Immediately after mixing, each reaction was split to allow for simultaneous reads in either a GloMax Discover Multimode Microplate Reader (Promega) or in the handheld luminometer. 200 µL of each reaction was transferred to a solid, white, nonbinding surface (NBS) 96-well plate (Costar) and read on a GloMax Discover Multimode Microplate Reader (Promega) collecting total luminescence using kinetic read over 1h. In parallel, the remaining 400 µL of each reaction remained in the swab jacket and was read with the handheld luminometer at 30min, 45min, and 1 h. To determine the correlation between the two instruments, the signals for each concentration of nucleocapsid protein were graphed and analyzed with linear regression.

1.5 Solution-Based SARS-CoV-2 RBD or N Immunoassay on a Handheld Luminometer.

A 4X master mix stock solution containing 120 ng/mL β9-labeled anti-SARS-CoV-2 spike RBD antibody D003, 240 ng/mL β10-labeled anti- SARS-CoV-2 spike RBD antibody D002, and 4 µM LgTrip protein was prepared in PBSB, and 100 µL/well was dispensed into the swab jacket. Either a 4X solution containing recombinant SARS-CoV-2 Spike RBD (refer to section 1.3 for inclusive list), heat-inactivated virus (refer to section 1.3 for inclusive list), or patient sample and 100 µL/ well was added to the swab jackets containing the master mix. Lastly, 200 µL/well of a 30-fold dilution of Fz (Nano-Glo Live Cell Substrate; Promega N205) in PBSB was added to the jackets. Assays were read on a standard handheld luminometer

by collecting total luminescence using kinetic or endpoint reads, depending on the experimental design. The same method was carried out for the nucleocapsid model system by using 120 ng/mL β9-labeled anti-SARS-CoV-2 N mAb Cat. 9547, 240 ng/mL β10-labeled anti-SARS-CoV-2 N mAb Cat. 9548, and 4 µM LgTrip and creating 4X titrations of recombinant CoV N proteins, heat-inactivated SARS-CoV-2 variants, or patient samples.

1.6 Testing the Effect of Transport Media on the SARS-CoV-2 N Immunoassay.

A 2X Master mix containing 60 ng/mL β9-labeled anti-SARS-CoV-2 N mAb Cat. 9547, 120 ng/mL β10-labeled anti-SARS-CoV-2 N mAb Cat. 9548, and 2 µM LgTrip in PBSB was made and 50 µL /well was added to a 96-well plate (Costar). A 2X 1:2 dilution series of various transport medias were spike with a constant 100 ng/ml SARS-CoV-2 N (Meridian Bioscience) in each dilution. This plate was incubated at ambient temperature for 90 min. After the incubation, 100 µL/well of 30-fold diluted Fz (Promega N205) was added and read on a GloMax.

The following viral transport medias were purchased for this work. Starswab II Bacterial Culture Collection and Transport System Cat. SP132 (Starplex), PrimeStore MTM Cat. 304255 (Longhorn Vaccine & Diagnostics), Puritan UniTranz-RT Transport System Cat. UT-100 (Puritan Medical Products Company), FlexTrans Transport Medium Cat. B1029-90D (Bartels Trinity Biotech), Viral Transport Media Cat. A48750DK (Gibco), Transport Medium Cat. 25-500-CI (Corning), Aptima Specimen Transfer Kit Cat. 301154C (Hologic).

1.7 Expression and Purification of β9 (Peptide 840) and β10 (HiBiT) Genetic Fusions with SARS-CoV-2 RBD Proteins.

HEK293/T cells were grown to approximately 70–90% confluency, then used to seed 2x 500mL spinner flasks with $1.25x10^8$ cells and recommended complete media plus 5 mL 1X pluronic F-68 polyol. Flasks were incubated at 37°C, 5% CO2 with stirring at 60–80rpm for 2–4 hours before transfection of plasmids expressing IL6-β9-HaloTag Linker-RBD-3xFlag or IL6-β10-15Gly/Ser linker-3XFlag with a CMV promoter made into DNA:PEI complexes. The DNA:PEI complexes were prepared for each flask by adding 400 µg DNA (0.8 µg/mL) for each RBD genetic fusion with a His Tag to 25 mL serum free media (SFM). In a separate tube, 1.2 mL ViaFect was added to 25 mL SFM. Tubes were mixed separately for 5 min at room temperature at 750 rpm. The DNA/SFM was added dropwise to the PEI/SFM, mixed gently by pipetting, and incubated at room temperature for 20 min to ensure proper complex formation. After incubation, 50 mL transfection complex was added to the 450 mL of cell culture. Spinner flasks were grown for 48 h at 37°C, 5% CO2 with stirring at 60–80 rpm. Media was collected and genetic fusion proteins were batch purified with ANTI-FLAG M2 Affinity Gel (Sigma).

1.8 Complete SARS-CoV-2 Serology Assay Lyophilization in Swab Jackets and Characterization.

The 4X stock assay reagent solution contained the following: 2.5% w/v Pullulan, 5 mM 6-Aza2-thiothymine (ATT), 5 mM ascorbic acid, 20 mM HEPES (pH 8.0), 90 mM Glycine, 20 mM Histidine, 25 mg/mL Sucrose, 0.01% Polysorbate 80, 240 ng/mL β10-labeled anti-SARS-CoV-2 RBD or N antibody (clone 505E 9A12 A3), 120 ng/mL β9-labeled anti- SARS-CoV-2 RBD or N antibody, 4 µM LgTrip 5146, and 40 µM Fz. All materials used to make the complete complex buffer were from Sigma. Aliquots (400 µL) were prepared in disposable swab jackets for

lyophilization. The swab jackets were partially capped prior to loading into the lyophilizer (Virtis Genesis 12EL) with shelves pre-set to 4 °C. Product then underwent a freezing step with a shelf temperature of -50 °C for 2 h. Upon evacuation of the system the lyophilization process was performed between shelf temperatures of -25 °C and 25 °C and pressures of 75 and 200 mTorr. The ice sublimation phase lasted 8 h and the bound water desorption phase lasted 16 h. At the end of the lyophilization process, the swab jackets were covered with a temporary stopper under atmospheric conditions.

1.9 Human Subjects Nasopharyngeal Swab and Serum Sample Collection.

SARS-CoV-2 positive serum samples (*n = 13*) were obtained from local participants who previously had a SARS-CoV-2 PCR+ test result but were in the convalescent stage. This study was approved by the Human Subjects Board of Promega Corporation and informed consent for participation in the study for purposes of clinical research was signed by the individuals. Following HIPPA compliance, all samples have been de-identified and assigned a new I.D. number. Additionally, SARS-CoV-2 positive nasopharyngeal swab samples (*n = 55)* and negative samples (*n = 21*) determined by the CDC 2019-Novel Coronavirus (2019-nCoV) Real-Time RT-PCR Diagnostic Panel test were collected by and obtained from the University of Wisconsin Hospital and Clinics, Clinical Laboratories, Madison, WI, United States. Following HIPPA compliance, all samples were deidentified. Another set (n=34) of positive SARS-CoV-2 nasopharyngeal swabs were obtained and collected from the Wisconsin State Lab of Hygiene. Following HIPPA compliance, these samples were collected with informed consent from the volunteer, and samples were deidentified before use in this study.

1.10 Commercially Sourced Serum Samples.

Commercially available negative serum samples from pre-pandemic timepoints from healthy individuals were sourced from Boca Biolistics (*n=12*).

1.11 Protein Quantification.

Protein concentration was determined by measuring the absorbance at 280 nm with a spectrophotometer (NanoDrop 8000, Thermo Fisher), following the manufacturer's instructions.

2 Supplementary Figures and Tables

2.1 Supplementary Figures

(A)

β9 (Peptide 840) Sequence:

GKLLFTVTIEKYK differences compared to peptide 245

GKLLFTVTIEKYK differences compared to peptide 521

(B)

LgTrip 5146 Sequence:

MKHHHHHHVFTLDDFVGDWEQTAAYNLDQVLEQGGVSSLLQNLAVSVTPIMRIVRSGENALKIDIHV
IIPYEGLSADQMAQIEEVFKVVYPVDDHHFKVILPYGTLVIDGVTPNKLNYFGHPYEGIAVFDGEKI
TTTGTLWNGNKIIDERLIDPD differences compared to LgTrip 3546

Supplementary Figure 1. (**A**) β9 (peptide 840) amino acid substitutions. Shown are amino acid substitutions between β9 (peptide 840) and previously reported β9 peptides, 245 shown in red and 521 shown in green. (**B**) LgTrip 5146 amino acid substitutions. Amino acid substitutions in LgTrip 5146 are highlighted in red compared to previously reported LgTrip 3546.

(A)

		Ab1-β9 ng/mL											
		250.000	125.000	62.500	31.250	15.625	7.813	3.906	1.953	0.977	0.488	0.244	0.000
Ab2 -β10, ng/mL		1	2	3	4	5	6	7	8	9	10	11	12
250.00	A												
125.00	B												
62.50	C												
31.25	D												
15.63	E												
7.81	F												
3.91	G												
0.00	H												

(B)

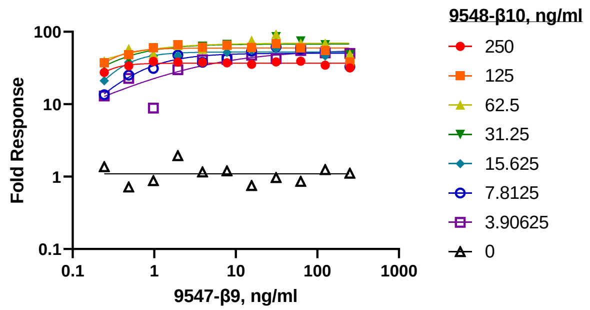

(C)

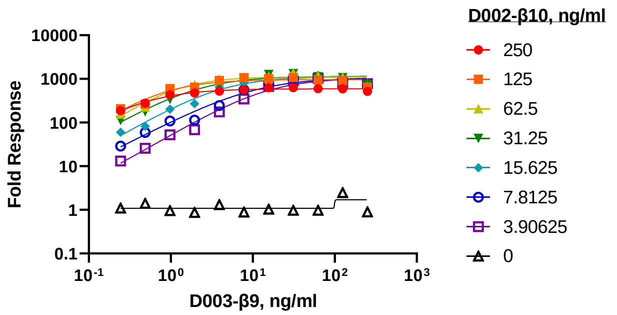

(D)

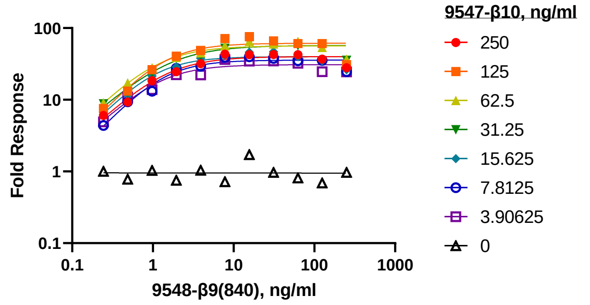

(E)

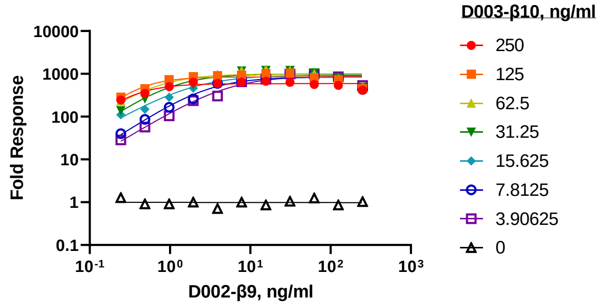

(F)

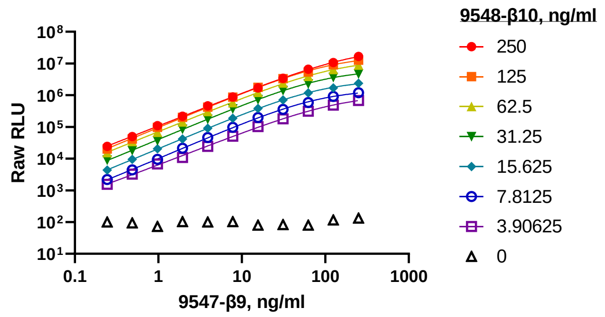

(G)

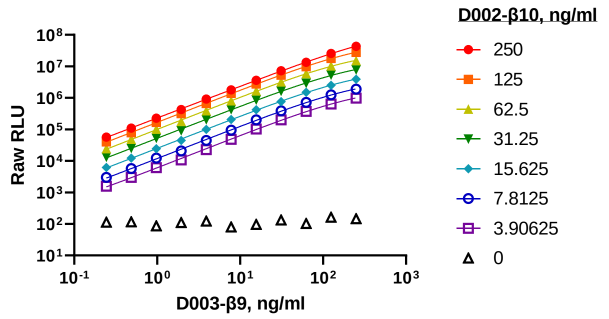

Supplementary Figure 2. Determining the optimal concentrations of the labeled antibodies. (**A**) Example matrix experiment of labeled antibodies being titered against one another across and down a 96-well plate in the presence or absence of antigen to determine optimal [Ab] that produce the largest fold response. (**B**) Fold response results from matrix experiment for SARS-CoV-2 nucleocapsid target antigen (50ng/ml). Optimal concentrations were determined to be 30 ng/mL β9-labeled anti-SARS-CoV-2 N mAb Cat. 9547 and 60 ng/mL β10-labeled anti-SARS-CoV-2 N mAb Cat. 9548. **(C**) Fold response results from matrix experiment for SARS-CoV-2 RBD target antigen. Optimal concentrations were determined to be 30 ng/mL β9-labeled anti-SARS-CoV-2 spike RBD antibody D003 and 60 ng/mL β10-labeled anti- SARS-CoV-2 spike RBD antibody D002. 1μM LgTrip was used in both experiments. (**D**) Fold response results from matrix experiment for opposite orientation of β9 or β10 labeled SARS-CoV-2 nucleocapsid antibodies. (**E**) Fold response results from matrix experiment for opposite orientation of β9 or β10 labeled SARS-CoV-2 spike RBD antibodies. (**F**) Raw RLU results from matrix experiment for SARS-CoV-2 nucleocapsid target antigen (50ng/ml) with the optimized peptide orientation. (**G**) Raw RLU results from matrix experiment for SARS-CoV-2 spike RBD target antigen (50ng/ml) with the optimized peptide orientation. Representative experiments shown here *(n=1*).

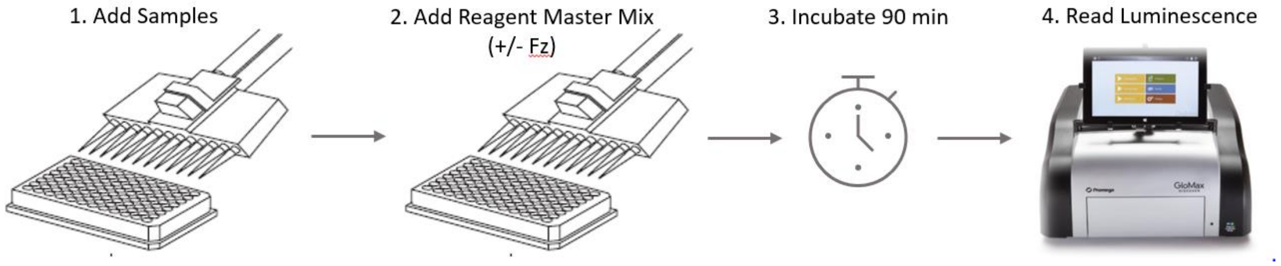

Supplementary Figure 3. Workflow for the plate-based SARS-CoV-2 antigen immunoassay utilizing the ternary Nluc reporter system. Samples are added to the 96-well plate (**1**) and then the 2x reagent master mix is added, which can either include Fz substrate or wait to add Fz post-incubation (**2**). The plate is covered and incubated for 90 minutes (**3**) and then read on a luminometer (**4**).

(A)

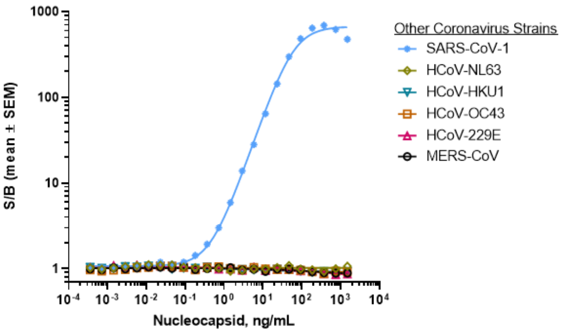

(B)

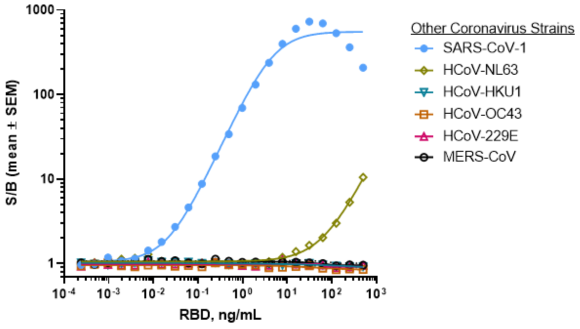

Supplementary Figure 4. Detection of purified proteins from other coronavirus strains. (**A**) Dose response curve of purified nucleocapsid proteins from strains using nucleocapsid antigen assay. Final antibody concentrations were 30 ng/ml β9-labeled anti-SARS-CoV-2 N mAb Cat. 9547 and 60 ng/mL β10-labeled anti-SARS-CoV-2 N mAb Cat. 9548. (**B**) Dose response curve of purified RBD proteins from strains using RBD antigen assay. Final antibody concentrations were 30 ng/mL β9-labeled anti-SARS-CoV-2 spike RBD antibody D003 and 60 ng/mL β10-labeled anti- SARS-CoV-2 spike RBD antibody D002. 1 μM LgTrip was used in all experiments. Reagents were incubated for 90 minutes prior to addition of substrate. Luminescence was measured and the signal over background (S/B) was calculated for each concentration of purified viral protein. Shown are means ± SEM (n = 3 independent experiments).

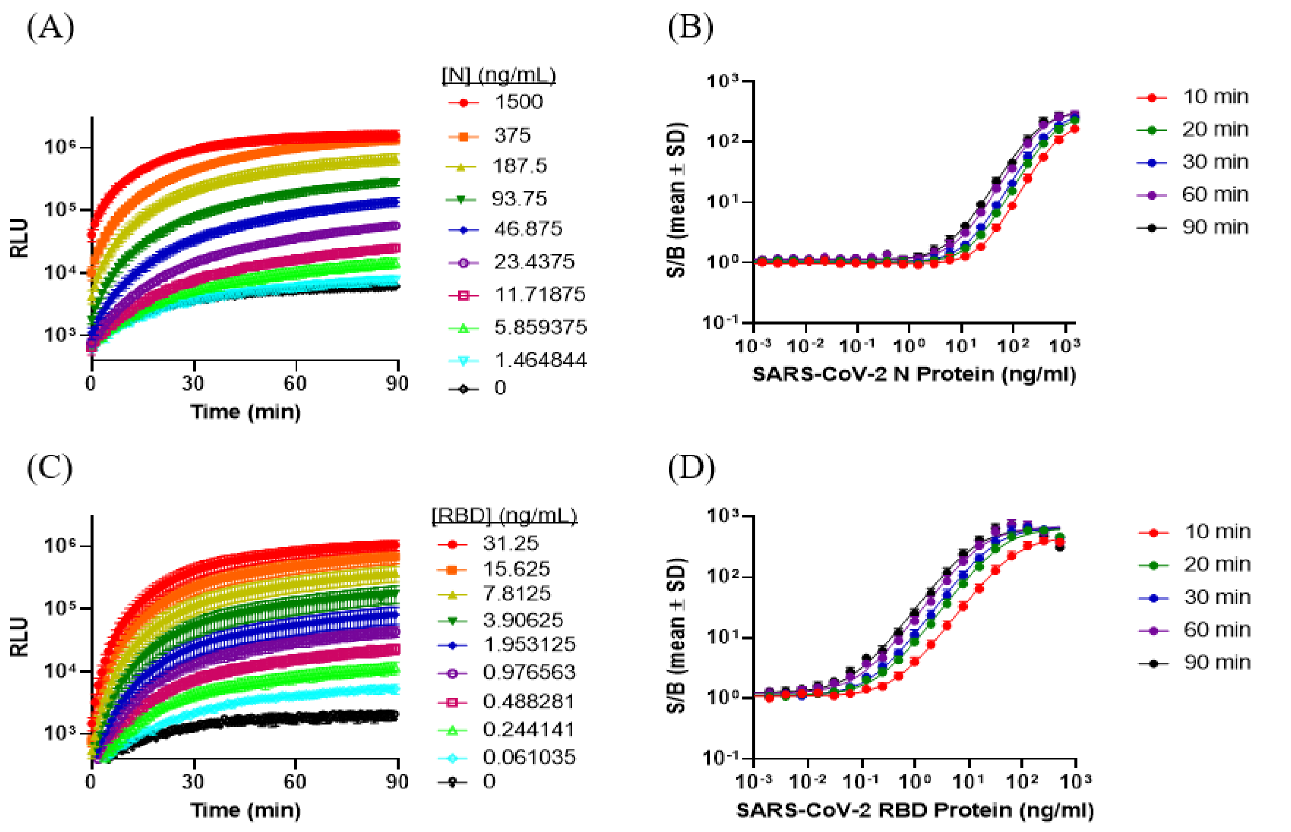

Supplementary Figure 5. Assay complexation kinetics. (**A**) Signal kinetics of all complementation pieces coming together with variable concentrations of purified SARS-CoV-2 nucleocapsid protein using nucleocapsid antigen assay (**B**) Conversion of the signal kinetic data in panel (a) to show dose response curves of purified nucleocapsid protein using nucleocapsid antigen assay. Final antibody concentrations were 30 ng/ml β9-labeled anti-SARS-CoV-2 N mAb Cat. 9547 and 60 ng/mL β10-labeled anti-SARS-CoV-2 N mAb Cat. 9548. (**C**) Signal kinetics of all complementation pieces coming together with variable concentrations of purified SARS-CoV-2 RBD protein using RBD antigen assay. (**D**) Conversion of the signal kinetic data in panel (c) to show dose response curves of purified RBD protein using nucleocapsid antigen assay. Final antibody concentrations were 30 ng/mL β9-labeled anti-SARS-CoV-2 spike RBD antibody D003 and 60 ng/mL β10-labeled anti- SARS-CoV-2 spike RBD antibody D002. For all experiments 1 µM LgTrip was used, Fz substrate was added in with the reagents. and luminescence was read kinetically for 90 minutes.

(A) (B)

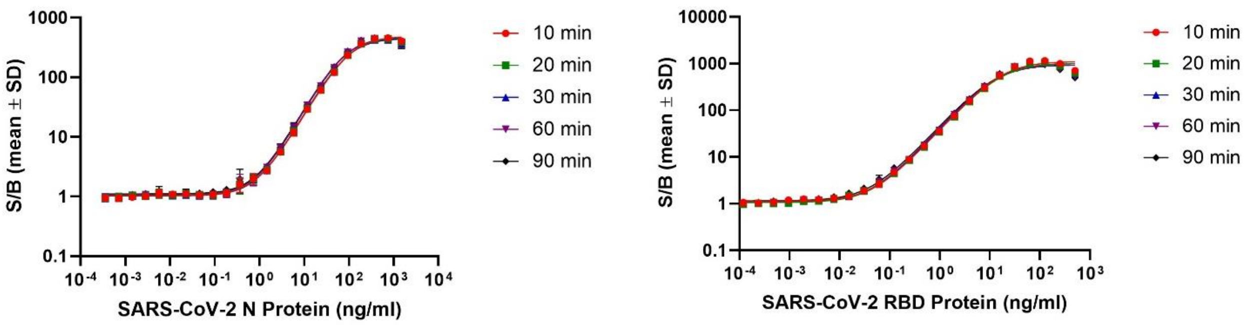

Supplementary Figure 6. Assay substrate consumption kinetics. (**A**) Signal kinetics of the glow reaction (substrate consumption) in a dose response curve of purified SARS-CoV-2 nucleocapsid protein using nucleocapsid antigen assay. Reagents were incubated for 90 minutes prior to addition of Fz substrate. Luminescence was read kinetically for 90 minutes. Final antibody concentrations were 30 ng/ml β9-labeled anti-SARS-CoV-2 N mAb Cat. 9547 and 60 ng/mL β10-labeled anti-SARS-CoV-2 N mAb Cat. 9548. (**B**) Signal kinetics of the glow reaction (substrate consumption) in a dose response curve of purified SARS-CoV-2 RBD protein using RBD antigen assay. Reagents were incubated for 90 minutes prior to the addition of Fz substrate. Luminescence was read kinetically for 90 minutes. Final antibody concentrations were 30 ng/mL β9-labeled anti-SARS-CoV-2 spike RBD antibody D003 and 60 ng/mL β10-labeled anti- SARS-CoV-2 spike RBD antibody D002. 1 μM LgTrip was used in all experiments. Luminescence was measured and the signal over background (S/B) was calculated for each concentration of purified viral protein. Shown are means ± SD of a representative experiment.

(A)

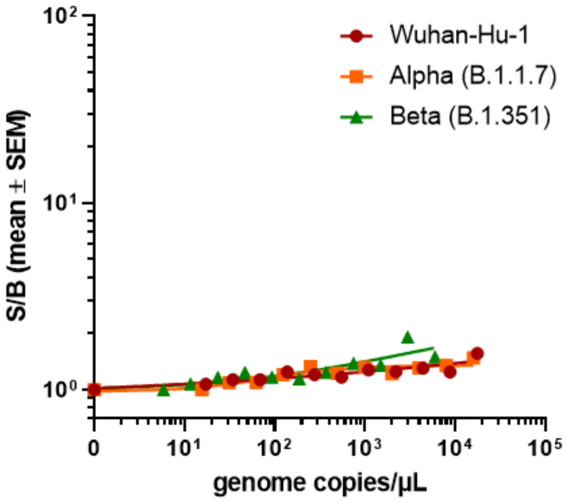

(B)

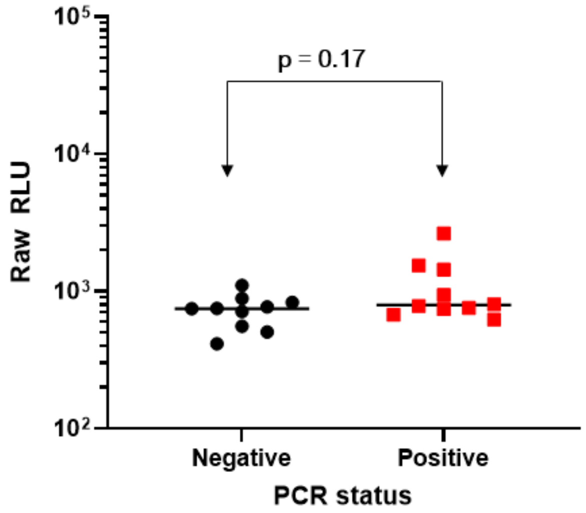

Supplementary Figure 7. RBD antigen assay performance with clinically relevant samples. (**A**) RBD antigen assay with SARS-CoV-2 heat-inactivated viruses. Luminescence was measured, and the signal over background (S/B) was calculated for each concentration of heat-inactivated virus. Shown mean ± SEM (*n* = *3* independent experiments). (**B**) Residual clinical nasopharyngeal smples that had previously been identified by PCR as negative (*n=10*) or positive (*n=10*) were individually tested in the RBD antigen assay. In both panels A and B, 30 ng/mL β9-labeled anti-SARS-CoV-2 spike RBD antibody D003, 60 ng/mL β10-labeled anti- SARS-CoV-2 spike RBD antibody D002, and 1μM LgTrip were incubated in the presence of sample for 90 min prior to the addition of Fz substrate and the measurement of luminescence. Representative experiments shown here. Statistical analyses determined by using an unpaired, non-parametric Mann-Whitney t-test.

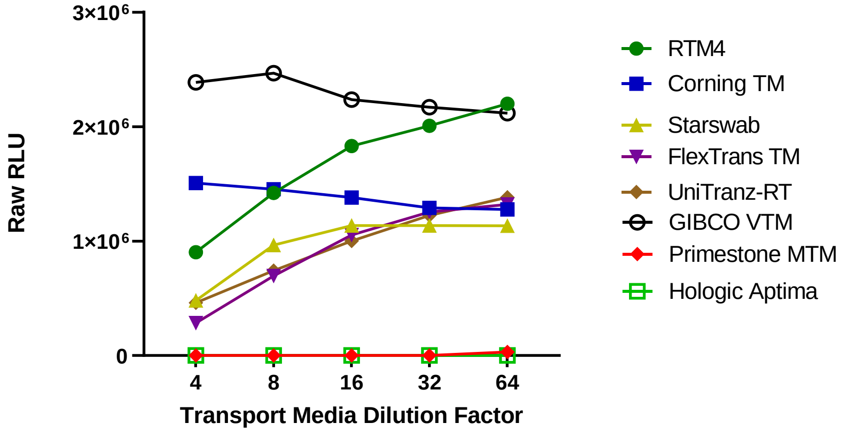

Supplementary Figure 8. The effect of viral transport media on the SARS-CoV-2 nucleocapsid immunoassay. Serially diluted commercially available mediums in the presence of 50ng/ml nucleocapsid protein. Final assay reagent concentrations were 30 ng/ml β9-labeled anti-SARS-CoV-2 N mAb Cat. 9547, 60 ng/mL β10-labeled anti-SARS-CoV-2 N mAb Cat. 9548, and 1 μM LgTrip, and 30-fold dilution of Fz with a 90-minute incubation prior to addition of Fz substrate. Luminescence was measured and raw RLU is shown.

(A)

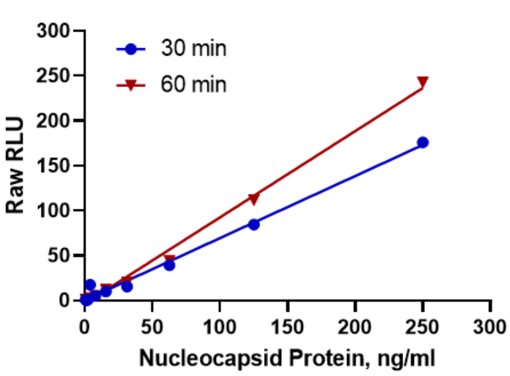

(B)

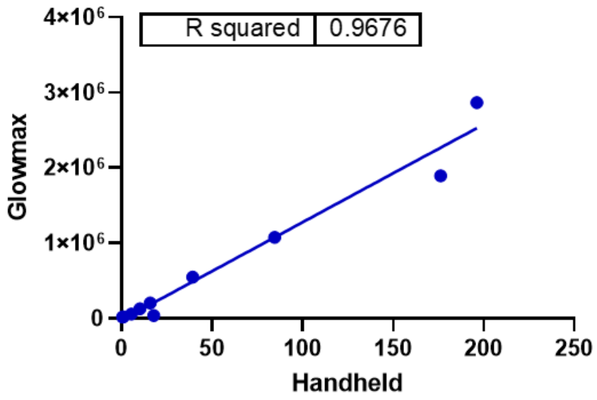

Supplementary Figure 9. N antigen assay detection with a handheld luminometer. (**A**) Dose response curve of purified nucleocapsid protein using nucleocapsid antigen assay with reads at 30- or 60-min. (**B**) Comparison of RLU values from handheld luminometer to GloMax plate reader for the same dose response curve at 30-min time point. In both panels A and B, 30 ng/ml β9-labeled anti-SARS-CoV-2 N mAb Cat. 9547, 60 ng/mL β10-labeled anti-SARS-CoV-2 N mAb Cat. 9548, 1 µM LgTrip, and 30-fold dilution of Fz substrate were added to sample. Luminescence was measured at a given time point and raw RLU is shown.

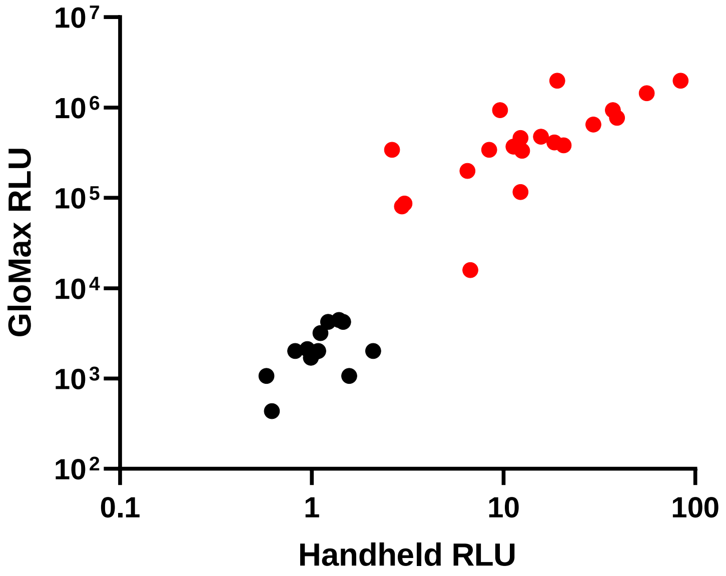

Supplementary Figure 10. Evaluation of SARS-CoV-2 antigen assay with nasal clinical samples. Clinical samples that had previously been identified by PCR as positive (*n=7*) or negative (*n=13*) were added either to the lyophilized or solution, plate-based N antigen assay then read with a handheld luminometer or GloMax after 60 min.

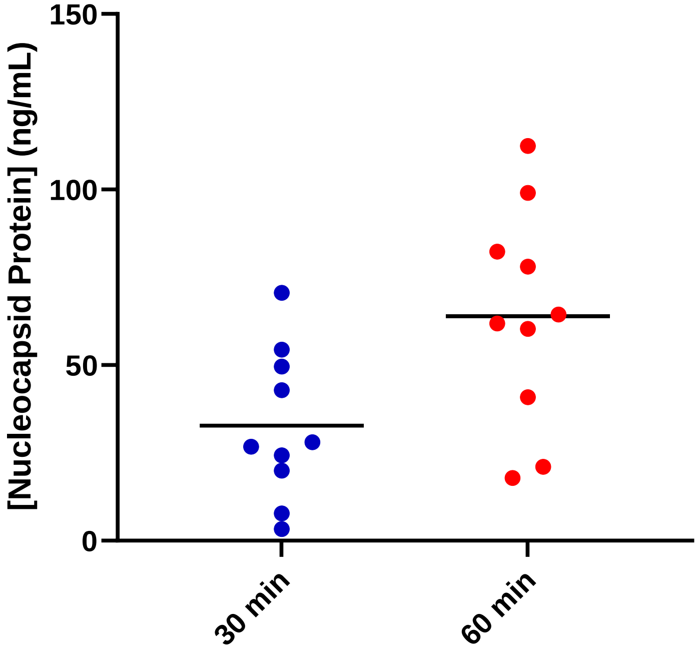

Supplementary Figure 11. Semi-quantitation of clinical samples read with handheld luminometer. Concentrations of nucleocapsid protein for PCR positive clinical samples (*n=10*) in Figure 5 were calculated by interpolation of the raw RLU values against the dose response curve for Wuhan Hu-1 purified nucleocapsid protein in Supplementary Figure 10A.

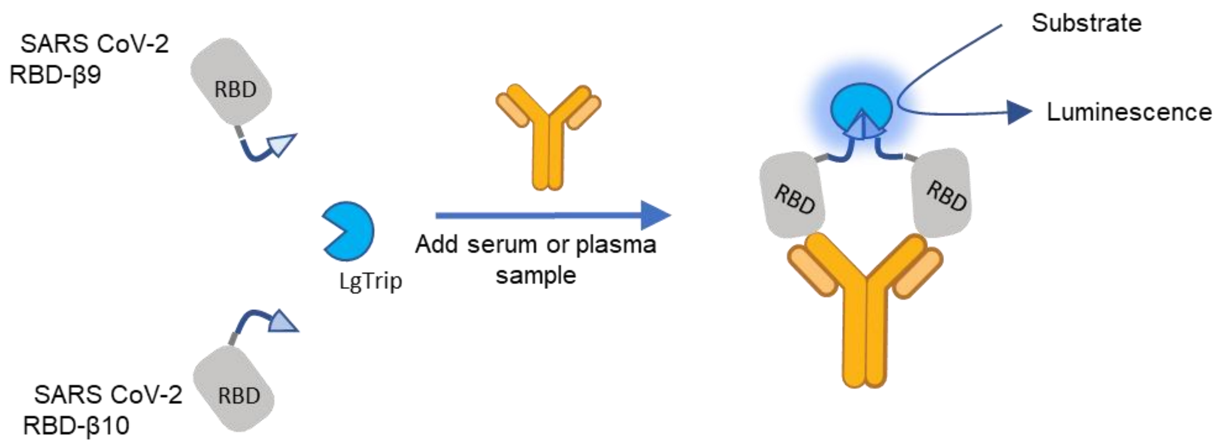

Supplementary Figure 12. Ternary Nluc-based assay for SARS-CoV-2 antibody response in serological samples. Schematic of the ternary Nluc reporter immunoassay utilizing RBD-β9 and RBD-β10 genetic fusion proteins as the affinity reagents. Binding to the SARS-CoV-2 antibodies in serum or plasma brings the RBD proteins and their respective peptide subunits into close proximity. With the addition of LgTrip and the furimazine substrate, complementation of the active ternary Nluc complex occurs producing bioluminescence.
